# Myocardial Fibroblast Activation After Acute Myocardial Infarction: A Positron Emission Tomography and Magnetic Resonance Study

**DOI:** 10.1016/j.jacc.2024.10.103

**Published:** 2025-02-18

**Authors:** Anna K. Barton, Neil J. Craig, Krithika Loganath, Shruti Joshi, Vasiliki Tsampasian, Menaka Mahendran, Joel Lenell, Evangelos Tzolos, Trisha Singh, Beth Whittington, Jennifer Nash, Michelle C. Williams, Edwin J.R. van Beek, Mark G. MacAskill, Bronwyn Berkeley, Stefan Vezaides, Mairi Brittan, Andrew H. Baker, Stephanie Sellers, Alison Fletcher, Tim Clark, Clint Waight, Riemer H.J.A. Slart, Daniel Berman, Damini Dey, Piotr Slomka, David E. Newby, Marc R. Dweck

**Affiliations:** a British Heart Foundation Centre of Research Excellence, the University of Edinburgh, Edinburgh, Scotland, United Kingdom; b Norwich Medical School, University of East Anglia, Norwich, United Kingdom; c Uppsala Clinical Research Center, Uppsala University, Uppsala, Sweden; d Department of Cardiology, Southampton General Hospital, University Hospital Southampton NHS Foundation Trust, Southampton, Hampshire, United Kingdom; e Edinburgh Imaging Facility, Queen’s Medical Research Institute, Edinburgh, Scotland, United Kingdom; f Department of Radiology and Centre for Heart Lung Innovation, St Paul’s Hospital and University of British Columbia, Vancouver, British Columbia, Canada; g NHS Lothian, The Royal Infirmary of Edinburgh, Edinburgh, Scotland, United Kingdom; h Medical Imaging Center, Department of Nuclear Medicine and Molecular Imaging, University Medical Center Groningen, Groningen, the Netherlands; i Departments of Medicine, Biomedical Sciences and Imaging, Cedars-Sinai Medical Center, Los Angeles, California, USA

**Keywords:** fibroblast activation protein inhibitor, molecular PET, myocardial Infarction

## Abstract

**Background:**

Myocardial fibrosis is a key healing response after myocardial infarction driven by activated fibroblasts. Gallium-68-labeled fibroblast activation protein inhibitor ([^68^Ga]-FAPI) is a novel positron-emitting radiotracer that binds activated fibroblasts.

**Objectives:**

The aim of this study was to investigate the intensity, distribution, and time-course of fibroblast activation after acute myocardial infarction.

**Methods:**

A total of 40 patients with acute myocardial infarction underwent hybrid [^68^Ga]FAPI-46 positron emission tomography and cardiac magnetic resonance and were compared with matched control subjects (n = 19) and those with chronic (>2 years) myocardial infarction (n = 20). Intensity of [^68^Ga]FAPI-46 uptake was quantified by maximum target-to-background ratio (TBR_max_). Burdens of fibroblast activation and scar were assessed by percent myocardial involvement of [^68^Ga]FAPI-46 uptake and late gadolinium enhancement, respectively.

**Results:**

Myocardial [^68^Ga]FAPI-46 uptake was observed in the acute infarct and peri-infarct regions that exceeded the extent of late gadolinium enhancement (burden 27.8% ± 12.4% vs 15.2% ± 10.6%; *P* < 0.001). One-third of patients also demonstrated right ventricular involvement. Myocardial [^68^Ga]FAPI-46 uptake was most intense at 1 and 2 weeks before declining at 4 and 12 weeks (TBR_max_ 4.0 ± 1.1, 3.7 ± 1.0, 3.1 ± 0.8, and 2.7 ± 0.7; *P* < 0.001). In comparison with control subjects, increased [^68^Ga]FAPI-46 uptake was observed in chronic (7 ± 6 years ago) infarcts at lower intensity than acute infarction (TBR_max_ 1.2 ± 0.1 vs 1.7 ± 0.5 vs 4.0 ± 1.1; *P* < 0.001). Baseline [^68^Ga]FAPI-46 burden correlated with lower left ventricular ejection fraction (*r* = −0.606), higher indexed left ventricular end-diastolic volume (*r* = 0.572), and higher scar burden (*r* = 0.871) at 1 year (*P* < 0.001 for all). Increased remote myocardial [^68^Ga]FAPI-46 uptake was associated with left ventricular dilatation and systolic dysfunction.

**Conclusions:**

Myocardial fibroblast activation peaks within a week of acute myocardial infarction and extends beyond the infarct region. It declines slowly with time, persists for years, and is associated with subsequent left ventricular remodeling. (PROFILE-MI–The FAPI Fibrosis Study; NCT05356923)

## Graphical Abstract

**Figure:**
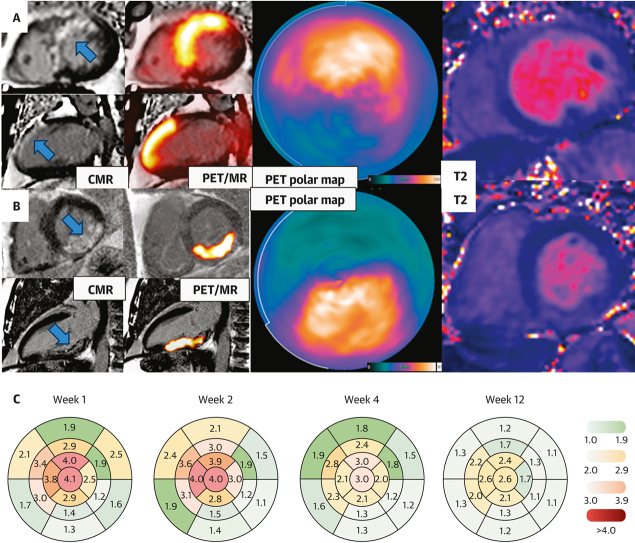

Coronary heart disease is the leading cause of death worldwide, with most of its morbidity and mortality attributable to acute myocardial infarction.^1^ Myocardial fibrosis is a fundamental healing response to the injury of myocardial infarction, with activated fibroblasts driving collagen deposition and fibrogenesis that aims to provide tissue integrity and reduce the risk of rupture. Although initially protective, dysregulated fibroblast activation and myocardial fibrosis activity may cause adverse remodeling and lead to the development of heart failure, arrhythmia, and major adverse cardiovascular events.^2^, ^3^, ^4^ The time-course of human fibroblast activation and myocardial fibrosis development after myocardial infarction remains unclear. Although generally considered complete by 3 months, this is largely based on preclinical data, with investigation in human myocardial infarction limited by a lack of appropriate methods for assessing fibroblast activation or fibrosis activity directly.^5^^,^^6^

The current clinical gold-standard method for the detection of myocardial fibrosis is cardiac magnetic resonance with late gadolinium enhancement and T1 mapping. However, this provides a measure of extracellular space expansion as a surrogate marker for fibrosis,^7^^,^^8^ and it cannot distinguish whether fibrotic processes are ongoing and modifiable or are established and irreversible. Moreover, expansion of the extracellular space is not exclusive to fibrosis and occurs in many disease processes, including edema and necrosis, which also characterize the early stages of acute myocardial infarction.

Fibroblast activation protein (FAP) is a membrane-bound serine protease expressed almost exclusively on activated fibroblasts.^9^, ^10^, ^11^ Its expression is not encountered in healthy adult tissue but is seen after myocardial infarction as well as in a range of other heart muscle diseases.^9^, ^10^, ^11^, ^12^ FAP inhibitors (FAPIs) have been developed and modified to facilitate positron emission tomography (PET) imaging of fibroblast activation and fibrosis activity.^13^ Although recent studies have confirmed the proof of principle that gallium-68-labeled FAP inhibitors (^68^Ga-FAPI) can be taken up in regions of infarcted myocardium, these have been limited to animal studies, retrospective and descriptive analyses, and small proof-of-concept studies without appropriate control subjects.^6^^,^^11^^,^^14^, ^15^, ^16^, ^17^, ^18^, ^19^ The time course of fibroblast activation in the weeks and months after acute myocardial infarction in humans remains unknown. In this prospective longitudinal cohort imaging study, we first investigated the time-course of fibroblast activation after acute myocardial infarction using hybrid [^68^Ga]FAPI-46 PET and magnetic resonance imaging, and we assessed its association with subsequent cardiac remodeling.

## Methods

### Clinical study design and study population

This prospective case-control longitudinal cohort study recruited participants aged >50 years with recent (<7 days) ST-segment elevation myocardial infarction from the Edinburgh Heart Centre, Edinburgh, Scotland, between April 2021 and March 2023. Participants with chronic myocardial infarction were recruited from outpatient clinics and review of records of patients admitted for treatment of ST-segment elevation myocardial infarction at least 2 years prior. Healthy control participants were recruited via email invitation sent to employees of the University of Edinburgh. Potential participants with an estimated glomerular filtration rate <30 mL/min/1.73 m^2^ or with contraindications to magnetic resonance imaging were excluded. The study was approved by the South-East Scotland Research Ethics Committee, and all participants provided written informed consent. The first author had unrestricted access to the data in the study and takes responsibility for its integrity and data analysis. The first and second authors (AKB and NC) drafted the manuscript, and all authors provided critical review.

Participants with acute myocardial infarction underwent either serial [^68^Ga]FAPI-46 PET and cardiac magnetic resonance at 1, 2, 4, and 12 weeks, or a single study within 4 weeks, with all invited back for repeated cardiac magnetic resonance with gadolinium contrast material at 12 months (Supplemental Figure 1). Participants with chronic myocardial infarction and age- and sex-matched control volunteers with no history of myocardial infarction, cardiomyopathy, or heart muscle disease underwent a single [^68^Ga]FAPI-46 PET and magnetic resonance study.

### Positron emission tomography and magnetic resonance imaging

Participants underwent imaging with a hybrid PET and magnetic resonance system (Biograph m-MR, Siemens Healthineers) with a 50-minute bedtime centered over the heart, 30 to 80 minutes after administration of 100 to 200 MBq [^68^Ga]FAPI-46. The magnetic resonance protocol is described in the Supplemental Materials. In short, after attenuation correction,^20^ the protocol also included late gadolinium enhancement images acquired after the administration of 1 mg/kg gadobutrol (Gadovist, Bayer Inc). Contrast imaging was not performed at weeks 2 and 4 in those patients undergoing serial magnetic resonance scans to limit contrast exposure.

### Image analysis

Image analysis was performed by expert readers (A.K.B., N.C., V.T., M.M., J.L., M.R.D.) blinded to group assignment and scan time. Magnetic resonance analysis was performed independently of the PET analysis using Circle Cardiovascular Imaging software. Left and right ventricular volumes and ejection fractions were measured using manual adjustment of automated contours on the short-axis cine stack. Left ventricular infarct size was quantified from late gadolinium enhancement sequences using the full-width half-maximum method to provide the myocardial infarct volume. Left ventricular infarct burden was then quantified as the myocardial infarct volume divided by the left ventricular myocardial volume, multiplied by 100 to be expressed as a percentage.

PET image analysis was performed using both a global and a regional approach to assess the intensity, volume, and burden of increased myocardial [^68^Ga]FAPI-46 uptake using 2 software platforms, QPET and FusionQuant, respectively (both Cedars Sinai). Both platforms allow precise image coregistration, automated motion correction, and advanced cardiac PET quantification.

#### Global myocardial [^68^Ga]FAPI-46 positron emission tomography analysis

Semiautomated segmentation of the myocardial PET signal arising from the left ventricle was performed in 3 orthogonal planes using an adjustable left ventricular region of interest as described previously.^21^ A mask was used to exclude all counts outside of the cardiac silhouette (Supplemental Figure 2).

The total volume of fibroblast activation was quantified based on pixels with [^68^Ga]FAPI-46 uptake above a conservative threshold of 1.5 × maximum blood pool activity, consistent with previous studies.^22^ This was used to calculate the burden of myocardial fibroblast activation (% of the myocardium with increased fibroblast activation) in an approach similar to that for the infarct burden (volume of increased myocardial [^68^Ga]FAPI-46 uptake divided by the total myocardial volume, multiplied by 100).

The intensity of myocardial [^68^Ga]FAPI-46 uptake was quantified using maximum and mean standard uptake values (SUV_max_ and SUV_mean_), both globally across the left ventricle and within individual segments of the American Heart Association 17-segment myocardial model.^23^ Maximum and mean target-to-background ratios (TBR_max_ and TBR_mean_) were calculated by correcting the myocardial uptake for the background blood pool activity measured in the right atrium.

#### Regional myocardial [^68^Ga]FAPI-46 positron emission tomography analysis

Analysis of the intensity of fibroblast activation across different regions of both the left and right ventricular myocardium was performed guided by magnetic resonance (Supplemental Figure 3). PET images were overlaid directly onto magnetic resonance short-axis late gadolinium enhancement sequences for all scans. First, the PET images and late enhancement sequences were fused in 3 orthogonal planes. Then, regions of interest were drawn using free-form polygons guided by the late gadolinium enhancement images, to measure tracer uptake in the infarct zone (defined as the area of late gadolinium enhancement), the peri-infarct zone (defined as areas of increased [^68^Ga]FAPI-46 signal [1.5 × blood pool signal]^22^^,^^24^ adjacent to the infarct zone but without underlying late gadolinium enhancement), and the remote myocardium (segment of normal myocardium without late gadolinium enhancement, diametrically opposite to the infarct on short-axis images according to the American Heart Association 17-segment model.^23^ Native T1 and T2 values were also measured in these infarct, peri-infarct, and remote myocardial areas. In addition, we identified areas of increased [^68^Ga]FAPI-46 uptake originating from the right ventricular myocardium and quantified this uptake by drawing free-form polygon regions of interest. SUV_max_ and TBR_max_ values were then calculated for all of the above regions of interest. In addition, and in order to estimate the effect of resolution and overspill of [^68^Ga]FAPI-46 signal from the myocardium into the blood pool, we measured the distance of PET uptake [1.5 × blood pool uptake³] extending radially from the region of scar defined by late gadolinium enhancement into the left ventricular blood pool (ie, signal where there is no underlying myocardium), as well as the distance extending circumferentially around the left ventricular myocardium in to the peri-infarct zone on the same short-axis slice (ie, signal where there is underlying myocardium forming a peri-infarct zone) on the first 20 scans from participants with acute myocardial infarction.

### Histologic analysis

Ex vivo assessment of FAP-positive fibroblasts in human myocardial infarction was performed using explanted myocardial tissue from patients with acute (<4 weeks; n = 3) and chronic (>2 years) myocardial infarction (n = 3) as well as healthy myocardium (n = 2) obtained at the time of orthotopic cardiac transplantation or autopsy. Tissue was obtained from a large tissue bank held at St Paul’s Hospital at the University of British Columbia under separate local ethical approval.

Histologic analysis is described in the Supplemental Material. Briefly, histologic analysis was performed on formalin-fixed, paraffin-embedded blocks of myocardial tissue using Masson’s trichrome staining performed to identify the infarct region, with an adjacent section undergoing immunohistochemistry of FAP expression.

### Statistical analysis

Statistical analyses of clinical characteristics and imaging data were performed using Prism version 10.2 (GraphPad). Comparisons between time points in the acute myocardial infarction group were assessed using 1-way analysis of variance. Multivariable linear regression to assess the factors influencing myocardial [^68^Ga]FAPI-46 uptake in patients with myocardial infarction was performed using backward stepwise selection initially incorporating age, sex, baseline ejection fraction, time since infarction in days, presence of comorbidities, and smoking status. Baseline and follow-up magnetic resonance parameters from the acute myocardial infarction group approximated a normal distribution and were compared using a paired Student’s *t-*test. A 2-sided *P* value of <0.05 denoted statistical significance.

## Results

### Study populations

Forty participants with acute ST-segment elevation myocardial infarction (age 61 ± 7 years; 15% female), 23 participants with chronic myocardial infarction 6.1 ± 5.4 years after their index infarction (age 69 ± 10 years; 22% female), and 21 control volunteers (age 59 ± 6 years; 33% female) were recruited (Table 1). Three participants with chronic myocardial infarction and 1 control volunteer withdrew because of claustrophobia, and 1 control volunteer was excluded after an area of basal septal noninfarct myocardial fibrosis was identified on the magnetic resonance scan. Thirty-five participants with acute myocardial infarction returned for follow-up imaging at 12 months.

**Table 1:** Study Population

	Acute MI (n = 40)	Chronic MI (n = 20)	Control Volunteers (n = 19)
Age, y	61 ± 7	69 ± 9	59 ± 6
Female, %	15	25	32
Ethnicity			
White	18	20	19
Black	1		
South Asian	1		
Body mass index, kg/m^2^	28 ± 4	28 ± 4	27 ± 6
Risk factors			
Hypercholesterolemia	32 (80)	11 (55)	2 (11)
Hypertension	11 (28)	6 (30)	5 (26)
Diabetes mellitus	4 (10)	3 (15)	0 (0)
Family history	9 (23)	3 (15)	2 (11)
Current or ex-cigarette smoker	27 (68)	12 (60)	5 (26)
Medications			
Aspirin	40 (100)	16 (80)	0 (0)
Dual-antiplatelet therapy	40 (100)	1 (5)	0 (0)
ACE inhibitor/ARB	36 (90)	17 (85)	4 (21)
β-blocker	34 (85)	10 (50)	1 (5)
Statin	38 (95)	20 (100)	5 (26)
Anticoagulation	0 (0)	4 (20)	0 (0)
Infarct characteristics			
Symptom to PCI time (mins)	403 ± 770 (38)	480 ± 1.325 (18)	–
Anterior	13 (33)	7 (35)	–
Inferior	20 (50)	10 (50)	–
Lateral	7 (18)	3 (15)	–

Values are mean ± SD, n, n (%), or mean ± SD (n), unless otherwise indicated.

ACE = angiotensin-converting enzyme; ARB = angiotensin receptor blocker; MI = myocardial infarction; PCI = percutaneous coronary intervention.

In total, 137 hybrid PET and cardiac magnetic resonance scans were performed in 80 participants. Of these, 97 scans were performed in 40 participants with acute myocardial infarction (27 at week 1, 30 at week 2, 21 at week 4, and 19 at week 12), 20 in participants with chronic myocardial infarction, and 20 in control volunteers (Supplemental Figure 1). Twenty of the 40 patients with acute myocardial infarction underwent the serial imaging study, and all of them underwent scans at week 1 and week 2, with 18 scanned at week 4 and 19 at week 12. Administration of [^68^Ga]FAPI-46 was well tolerated, with no participants reporting any immediate or short-term adverse reactions.

### Qualitative assessment of myocardial [^68^Ga]FAPI-46 uptake

**Figure 1:**
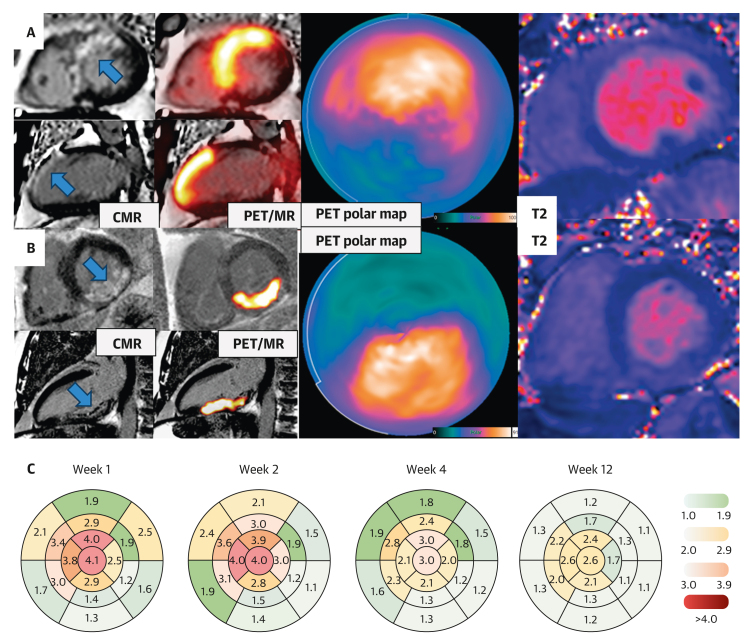
Regional Fibroblast Activation After Acute Myocardial Infarction (A, B) Cases of acute myocardial infarction (imaged 23 days (A) and 3 days (B) after myocardial infarction) affecting the anterior (A) and inferior (B) walls. Short-axis and 2-chamber magnetic resonance views are shown fused with PET. Late gadolinium enhancement is evident in the anterior (A) and inferior (B) walls, respectively (blue arrows). Intense [^68^Ga]FAPI-46 activity (TBR_max_ = 3.80 [A], 6.30 [B]) is observed in the infarct zone but extends into the peri-infarct region. Matched polar maps demonstrate increased anterior and inferior [^68^Ga]FAPI-46 signals, respectively, and are presented alongside the matched T2-weighted short-axis images. (C) TBR_max_ within each myocardial segment for the acute anterior myocardial infarcts are presented across time. Emboldened values seen in segments 7-9, 13-15, and 17 demonstrate the myocardial segments with a statistically significant change over time (*P* < 0.05). [^68^Ga]FAPI-46 = ^68^Gallium-fibroblast activation protein inhibitor-46; CMR = cardiac magnetic resonance; PET = positron emission tomography; TBR_max_ = maximal target-to-background ratio.

**Figure 2:**
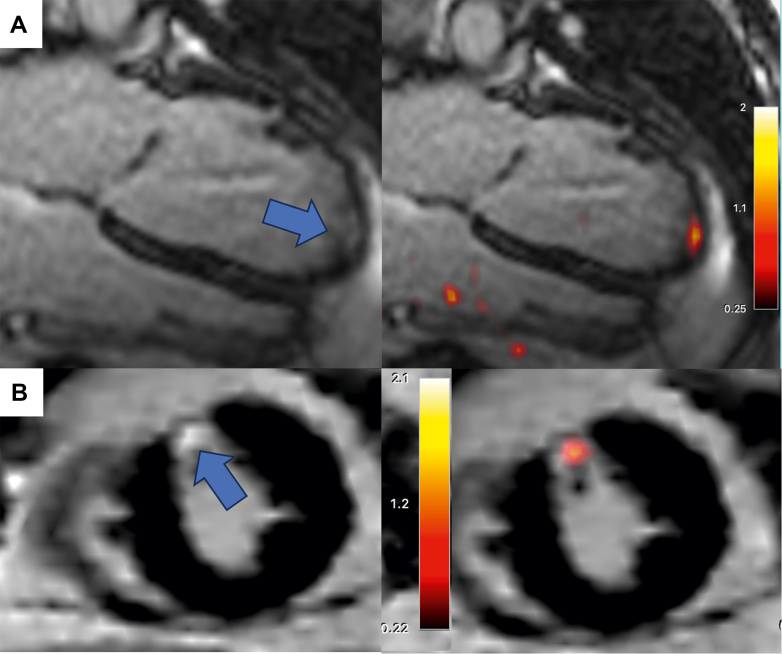
Low-Intensity [^68^Ga]FAPI-46 Uptake in Chronic Myocardial Infarction Two cases of chronic myocardial infarction are demonstrated. Long-axis (A) and short-axis (B) magnetic resonance views are shown alone (left) and fused with PET (right). Late gadolinium enhancement is evident in the apical (A) and mid anterior (B) walls, respectively (blue arrows). Case A was imaged 2.5 years and case B imaged 20 years after index myocardial infarction. An area of increased fibroblast activation is detected by [^68^Ga]FAPI-46 that is confined to the infarct zone, with no evidence of increased activity in the peri-infarct zone or remote myocardium. This activity is also of lower intensity than that observed in acute infarcts (TBR_max_ 1.8 ± 0.5 vs 3.8 ± 1.0, *P* < 0.001). Abbreviations as in Figure 1.

Qualitative visual assessment demonstrated focal infarct-related myocardial [^68^Ga]FAPI-46 uptake in all patients after acute myocardial infarction (Table 2, Figure 1). This was observed at all time points apart from 2 participants who had no visible [^68^Ga]FAPI-46 uptake at week 12. Modest areas of increased myocardial [^68^Ga]FAPI-46 uptake were apparent in 14 of 20 patients with chronic myocardial infarction (Table 2, Figure 2) and in 1 of the control volunteers, who was excluded because of demonstrable focal myocardial late gadolinium enhancement.

**Table 2:** Global Gallium-68-Fibroblast Activation Protein Inhibitor-46 Positron Emission Tomography and Magnetic Resonance Analysis

	Acute MI (Combined Single- and Multi-Timepoint Participants)				*P* Value for Trends Across Weeks 1, 2, 4, 12	Chronic MI (>24 Months) (n = 20)	Control Volunteers (n = 19)	*P* Value Chronic Myocardial Infarction vs Control Volunteers
	Week 1 (n = 27)	Week 2 (n = 30)	Week 4 (n = 21)	Week 12 (n = 19)				
Time since infarct	8 ± 2 d	15 ± 2 d	32 ± 10 d	88 ± 9 d	–	7 ± 6 y	–	–
Magnetic resonance imaging								
LVEF, %	58 ± 8	57 ± 9	56 ± 9	60 ± 6	0.60	59 ± 8	67 ± 5	**<0.01**
Indexed LVEDV, mL/m^2^	79 ± 14	79 ± 14	81 ± 21	78 ± 15	0.80	75 ± 14	73 ± 12	0.60
Infarct burden, % LGE	15.2 ± 10.6	–	–	10.3 ± 6.1	0.07	8.1 ± 7.2	0	**<0.01**
[^68^Ga]FAPI-46 positron emission tomography								
Visually positive myocardial [^68^Ga]FAPI-46	27 (100)	30 (100)	21 (100)	17 (89)	–	14 (70)	0 (0)	–
Intensity of fibroblast activation (TBR_max_)	4.0 ± 1.1	3.7 ± 1.0	3.1 ± 0.8	2.7 ± 0.7	**<0.001**	1.7 ± 0.5	1.2 ± 0.1	**<0.001**
Burden of fibroblast activation (% myocardium with increased [^68^Ga]FAPI-46)	27.8 ± 12.4	23.0 ± 14.4	19.6 ± 14.6	12.8 ± 8.1	**<0.01**	0.6 ± 2.2	0	0.26

Values are mean ± SD or n (%). **Bold***P* values are statistically significant.

[^68^Ga]FAPI-46 = gallium-68 fibroblast activation protein inhibitor-46; LVEDV = left ventricular end diastolic volume; LVEF = left ventricular ejection fraction; MI = myocardial infarction; PET = positron emission tomography; TBR_max_ = maximum target to background ratio; LGE = late gadolinium enhancement.

### Quantitative assessment of myocardial [^68^Ga]FAPI-46 uptake

#### Patients with acute MI

**Figure 3:**
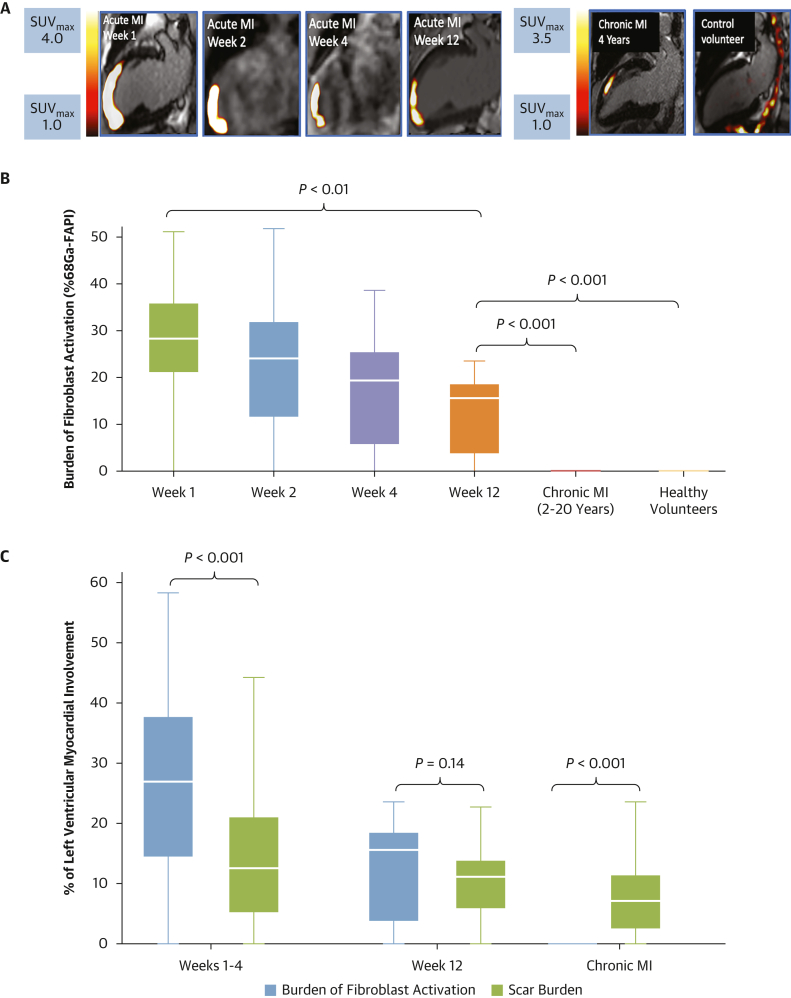
Burden of Myocardial Fibroblast Activation Over Time (A) Illustrative PET/MR images representing acute infarction at each time point are taken from the same participant. (B) Percentage of myocardium with increased [^68^Ga]FAPI-46 uptake demonstrating a stepwise reduction with time after acute infarction, with only relatively small areas of increased activity observed in chronic infarction. (C) In the initial stages of acute infarction, burden of fibroblast activation greatly exceeds the scar burden, forming a peri-infarct region. By 12 weeks, the burden of fibroblast activation and scar burden are similar. In chronic infarction, the burden of fibroblast activation is smaller than the area of late enhancement (C: weeks 1-4 include data only from the first scan from all 40 participants with acute infarction). (B and C: median ± IQI.) MI = myocardial infarction; other abbreviations as in Figure 1.

**Figure 4:**
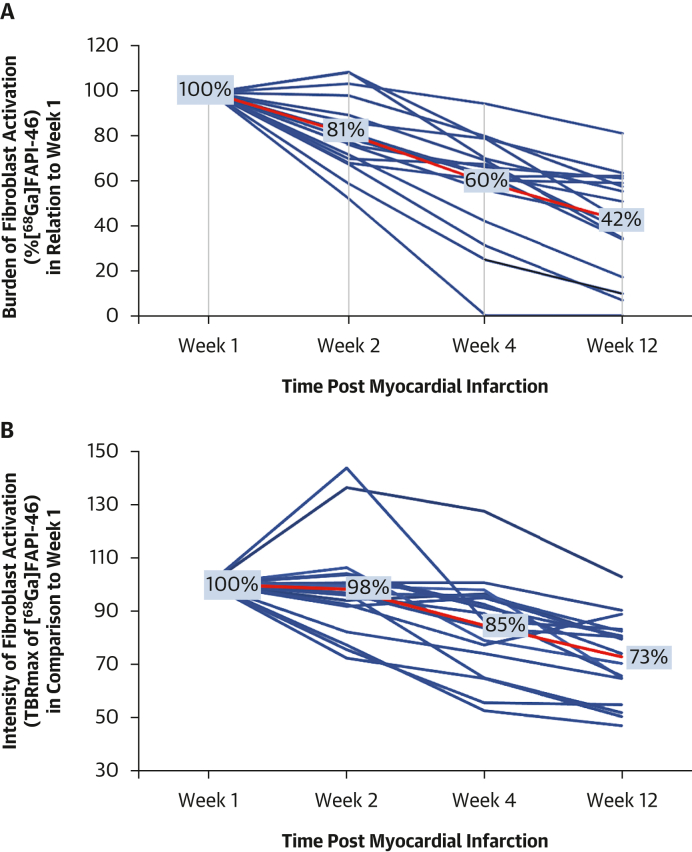
Burden and Intensity of Fibroblast Activation Over Time at the Individual Patient Level Twenty participants underwent serial combined [^68^Ga]FAPI-46 PET and MR at weeks 1, 2, 4, and 12 after myocardial infarction, 18 of whom underwent all 4 scans (1 participant missed weeks 4 and 12, and 1 missed week 4). Burden (%[^68^Ga]FAPI-46 [A]) and intensity (TBR_max_ [B]) of [^68^Ga]FAPI-46 activity declined in similar stepwise patterns over the 12 weeks, with the largest decline seen in burden of fibroblast activation, with less than one-third of the original volume involved at 3 months. Intensity declined by 25% of the baseline [^68^Ga]FAPI-46 uptake at week 1. Abbreviations as in Figure 1.

Both the burden and intensity of left ventricular myocardial [^68^Ga]FAPI-46 uptake were highest at 1 and 2 weeks after myocardial infarction, followed by a stepwise decline at 4 and 12 weeks (*P* < 0.001) (Table 2, Figure 3, Supplemental Figure 4, Supplemental Tables 1, 2, 3A-3F). These results were mirrored when only those undergoing multiple PET/magnetic resonance examinations at 1, 2, 4, and 12 weeks were considered (Figure 4). At week 1, the burden of myocardial [^68^Ga]FAPI-46 uptake was 183% greater than the burden of infarction identified by late gadolinium enhancement (27.8% ± 12.4% vs 15.2% ± 10.6%; *P* < 0.001). Regional analysis demonstrated that [^68^Ga]FAPI-46 uptake was observed not only in the acute infarct region but also in the peri-infarct zone (Table 3). There were no discernible differences in the intensity or burden of fibroblast activation between the territories (anterior, inferior, and lateral) of myocardial infarction, in contrast to scar burden, which was higher in anterior and lateral infarcts compared with inferior infarcts (23.7% ± 11.9% in anterior and 25.3% ± 10.1% in lateral vs 8.6% ± 6.4% in inferior infarction; *P* < 0.001). By 12 weeks, the burden of both late gadolinium enhancement and myocardial [^68^Ga]FAPI-46 uptake had reduced, with the burden of [^68^Ga]FAPI-46 uptake now similar to the burden of late gadolinium enhancement (12.8% ± 8.1% vs 10.3% ± 6.1%; *P* = 0.14) (Figure 3). Myocardial [^68^Ga]FAPI-46 intensity within the peri-infarct zone was lower than within the infarct zone at all time points. The mean distance of [^68^Ga]FAPI-46 uptake that overspilled radially from the myocardium into the left ventricular blood pool (6.1 ± 3.6 mm) was one-third of the mean circumferential distance that went beyond the region of late gadolinium enhancement and into the peri-infarct zone of the left ventricular myocardium (15.4 ± 5.4 mm; *P* < 0.001).

**Table 3:** Regional Gallium-68-Fibroblast Activation Protein Inhibitor-46 Positron Emission Tomography and Magnetic Resonance Analysis

	Time Point Post MI				
	Acute MI Weeks 1-4^a^ (n = 40)	Acute MI Week 12 (n = 19)	*P* Value Weeks 1-4 vs Week 12	Chronic MI >24 Months (n = 20)	*P* Value Week 12 vs Chronic MI
Time since infarct	11 ± 5 d	88 ± 9 d	–	7.1 ± 6 y	–
Infarct zone					
Visually increased [^68^Ga]FAPI-46 activity in infarct zone	40 (100)	17 (89)	–	14 (70)	–
Intensity of infarct zone [^68^Ga]FAPI-46 uptake, TBR_max_^b^	4.1 ± 1.1	3.0 ± 0.7	<0.001	1.9 ± 0.5	<0.001
Native T1 values, ms	1,498 ± 112	1,412 ± 150	0.004	1,450 ± 118	0.13
Native T2 values, ms	53 ± 8	47 ± 7	<0.05	48 ± 9	0.59
Peri-infarct zone					
Visually increased [^68^Ga]FAPI-46 activity in peri-infarct zone	37 (95)	15 (79)	–	1 (5)	–
Burden of peri-infarct [^68^Ga]FAPI-46 uptake, %^b^	12.1 ± 8.7	2.8 ± 4.0	<0.001	3.5^c^	–d
Intensity of peri-infarct zone [^68^Ga]FAPI-46 uptake, TBR_max_^b^	3.3 ± 0.8	2.7 ± 0.4	0.01	2.5^c^	–d
Native T1 values, ms	1,315 ± 89	1,263 ± 49	0.002	1,273 ± 58	0.57
Native T2 values, ms	46 ± 5	42 ± 4	<0.05	42 ± 3	0.83
Remote myocardium					
Visually increased [^68^Ga]FAPI-46 activity in remote myocardium	1 (3)	0 (0)	–	0 (0)	–
Intensity of remote myocardium [^68^Ga]FAPI-46 uptake, TBR_max_	1.0 ± 0.1	1.0 ± 0.1	0.52	1.0 ± 0.2	0.98
Native T1 values, ms	1,188 ± 84	1,183 ± 50	0.34	1,176 ± 84	0.99
Native T2 values, ms	40 ± 3	39 ± 2	0.44	39 ± 3	0.95
Right ventricle					
Visually increased [^68^Ga]FAPI-46 uptake in right ventricle	15 (38)	2 (11)	–	0 (0)	–
Intensity of right ventricular [^68^Ga]FAPI-46 uptake, TBR_max_^b^	2.2 ± 0.5	2.0 ± 0.1	0.43	n/a	–

Values are mean ± SD or n (%). **Bold***P* values are statistically significant.

n/a = not applicable; other abbreviations as in Tables 1 and 2.

a The participant’s first scan post myocardial infarction used for analysis.

b Values presented from only those participants with visually increased [^68^Ga]FAPI-46 uptake.

c SDs not provided because n = 1.

d Statistical comparisons not performed as n = 1.

Primary percutaneous coronary intervention was performed in 93% of the participants with acute myocardial infarction (Table 1). There was no difference in the [^68^Ga]FAPI-46 intensity (TBR_max_ 3.9 ± 1.2 vs 3.9 ± 1.2; *P* = 0.96) or burden (%FAPI 31% ± 16% vs 26% ± 15%; *P* = 0.38) of the 8 participants with the longest symptom onset to balloon time (1,241 ± 1,475 min) when compared with the remaining 29 participants (184 ± 80 min).

**Figure 5:**
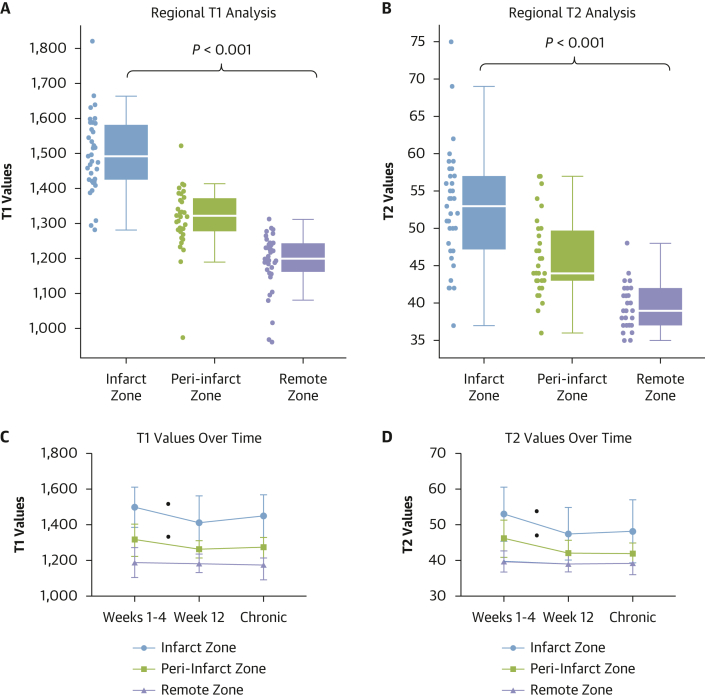
Regional T1 and T2 Analysis Over Time (A, B) Significant difference in T1 and T2 values between the infarct, peri-infarct. and remote zones of all contrast-enhanced MR scans conducted in the acute infarction cohort (*P* < 0.001). (C, D) Change in T1 and T2 values within each region over time. There is a significant decline in both T1 and T2 signal in the infarct and peri-infarct zones between weeks 1-4 and week 12 (all *P* < 0.05), but there was no change over time in the remote myocardium or in any zone between week 12 and chronic established myocardial infarction. Abbreviations as in Figure 1.

There was a positive association between segmental longitudinal strain and segmental [^68^Ga]FAPI-46 intensity (r = 0.312; *P* < 0.001). Similar to the ^68^Ga-FAPI uptake, T1 (1,498 ± 112 ms vs 1,315 ± 89 ms vs 1,188 ± 84 ms; *P* < 0.001) and T2 (53 ± 8 ms vs 46 ± 5 ms vs 40 ± 3 ms; *P* < 0.001) values were higher in both the infarct and peri-infarct zones compared to the remote myocardium. The T1 and T2 values decreased across the first 12 weeks in the infarct and peri-infarct zones (Table 3). In contrast to the [^68^Ga]FAPI-46 intensity and burden, patients at 12 weeks after acute infarction had similar myocardial T1 and T2 values to patients with chronic myocardial infarction (Table 3, Figure 5).

Myocardial [^68^Ga]FAPI-46 uptake remote from the site of infarction was of much lower intensity than the infarcted and peri-infarct regions (Table 3). However, notable increases in the remote myocardial [^68^Ga]FAPI-46 intensity were observed in 3 patients (TBR_max_ 1.3 ± 0.2 vs 1.0 ± 0.1; *P* < 0.001), all of whom had large infarcts (burden of late gadolinium enhancement 33% ± 12%) with marked left ventricular systolic dysfunction (ejection fraction 33% ± 6%) and adverse left ventricular remodeling (indexed end-diastolic volume of 117 ± 31 mL/m^2^). Indeed, the intensity of fibroblast activation in the remote myocardium across all patients correlated with a lower left ventricular ejection fraction (*r* = −0.575; *P* < 0.001) and higher indexed left ventricular end-diastolic volume (*r* = 0.636; *P* < 0.001).

Of the 40 participants with acute myocardial infarction, 15 patients (38%) demonstrated increased right ventricular myocardial [^68^Ga]FAPI-46 uptake which was more than double the uptake observed in the normal right ventricular myocardium (TBR_max_ 2.2 ± 0.5 vs 1.0 ± 0.2 respectively; *P* < 0.001) but lower than in regions of the adjacent left ventricular infarct (TBR_max_ 2.2 ± 0.5 vs 3.8 ± 0.9 respectively; *P* < 0.001). These 15 patients represented 60% of all patients with acute inferior infarction, and only 4 of these patients had clear evidence of right ventricular late gadolinium enhancement on their magnetic resonance scan (Table 3, Supplemental Figure 5). In the 9 participants with right ventricular [^68^Ga]FAPI-46 uptake who underwent a repeat scan at 12 weeks, only 2 participants had persistent right ventricular uptake.

### Patients with chronic myocardial infarction

Patients with chronic myocardial infarction had similar scar burden (8.1% ± 7.2% vs 10.3% ± 6.1%; *P* = 0.22) and left ventricular systolic function (left ventricular ejection fraction, 59.2% ± 8.3% vs 59.5% ± 6.4%; *P* = 0.91) compared to patients with acute myocardial infarction at the 12-week time point. Myocardial [^68^Ga]FAPI-46 uptake values were higher in patients with chronic infarcts than control volunteers, but lower than patients with acute myocardial infarction at 1 week (1.7 ± 0.5 vs 1.2 ± 0.1 vs 4.0 ± 1.1; *P* < 0.001) (Table 2, Supplemental Figure 4). Myocardial [^68^Ga]FAPI-46 uptake was confined within the infarct zone, with the burden of uptake being >10× smaller than the burden of chronic infarction identified by late gadolinium enhancement (0.6% ± 2.2% vs 8.1% ± 7.2%, respectively; *P* < 0.001).

### Determinants of myocardial [^68^Ga]FAPI-46 uptake

Across all patients with myocardial infarction, moderate associations were observed between the time from myocardial infarction and both the intensity (TBR_max_ *r* = −0.649; *P* < 0.001) and the burden (*r* = −0.631; *P* < 0.001) of myocardial [^68^Ga]FAPI-46 uptake, although increased fibroblast activation was still observed in a chronic infarct >20 years after the index event (TBR_max_ 2.0).

In a multivariable model incorporating both imaging and clinical variables, time from myocardial infarction remained the only predictor of the intensity of myocardial [^68^Ga]FAPI-46 uptake (*P* < 0.05). Scar burden (*P* < 0.05) and time from myocardial infarction (*P* < 0.05) were the only independent predictors of the burden of fibroblast activation in those with acute myocardial infarction.

### 1-year follow-up

Thirty-five participants with acute myocardial infarction underwent follow-up cardiac magnetic resonance imaging at 12 months (Supplemental Table 4), although 2 scans were excluded because of scanner failure or poor image quality. Across those patients, there was a reduction in the left ventricular infarct burden (15.4% ± 12.0% vs 10.5% ± 7.5%; *P* < 0.001) and an improvement in the indexed left ventricular end-diastolic volume (82.0 ± 18.9 mL/m^2^ vs 77.6 ± 15.4 mL/m^2^; *P* < 0.05), with an apparent trend for an improvement in left ventricular ejection fraction (56.5% ± 10.8% vs 58.3% ± 9.1%; *P* = 0.08).

The burden of myocardial [^68^Ga]FAPI-46 uptake at the baseline visit of the index acute myocardial infarction was associated with lower left ventricular ejection fraction (*r* = −0.606; *P* < 0.001), higher indexed left ventricular end-diastolic volume (*r* = 0.572; *P* < 0.001), and higher scar burden (*r* = 0.871; *P* < 0.001) at 12 months. Similarly, the peri-infarct [^68^Ga]FAPI-46 burden at baseline was also associated with subsequent lower left ventricular ejection fraction (*r* = −0.37; *P* < 0.05) and increased scar burden at 12 months (*r* = 0.49; *P* < 0.05).

[^68^Ga]FAPI-46 intensity in the remote myocardium was associated with a lower left ventricular ejection fraction (*r* = −0.605; *P* < 0.001) with a trend toward a higher left ventricular end-diastolic volume (*r* = −0.326; *P* = 0.056) at 12 months.

### Histology and immunohistochemistry

No FAP expression was seen in the samples of normal healthy myocardial tissue. By contrast, increased FAP expression was observed in both acute and chronic myocardial infarcts, although the staining was more intense in patients with acute infarction. In keeping with the in vivo data, FAP was observed in both the peri-infarct region and the remote myocardium of these patients with large myocardial infarcts (Supplemental Figure 6).

## Discussion

In this first longitudinal study using [^68^Ga]FAPI-46 in patients after myocardial infarction, we demonstrate intense fibroblast activation occurring rapidly after acute myocardial infarction, extending beyond the infarct into the peri-infarct zone and into the right ventricle in many cases. Fibroblast activation was also observed in the remote myocardium in patients who had sustained the largest infarcts. Interestingly, the burden of myocardial fibroblast activation in the acute phase correlated with infarct size, ventricular remodeling, and systolic function at 1 year. Ongoing fibroblast activation could still be observed within the infarct many years after the index event, indicating continuous and persistent long-term myocardial healing, ventricular remodeling, and scar maintenance. These novel findings have major implications for our understanding of the intensity, distribution, and time course of myocardial healing after myocardial infarction.

Previous studies investigating the role of fibroblasts in myocardial infarction have been limited to animal and histologic studies, retrospective analyses in humans, and small uncontrolled proof-of-concept descriptive ^68^Ga-FAPI PET studies.^6^^,^^11^^,^^14^, ^15^, ^16^, ^17^, ^18^, ^19^ The only study to evaluate the time course of fibroblast activation was a serial ^68^Ga-FAPI study in a rat model of acute myocardial infarction. This study suggested that fibroblast activation reached an early peak at day 6 before rapidly reducing back to baseline by 2 weeks.^6^ We have demonstrated that these cells driving adverse remodeling are activated and are at their most intense in humans within the first week of infarction, and that intense fibroblast activation remains present out to 3 months, with lower levels of activation observed many years or even decades later. Our observations confirm that fibroblast activation is not a feature of healthy myocardium and suggest that activated fibroblasts play a key long-term role in the healing response to, and scar maintenance of, myocardial infarction. More fundamentally, this study demonstrates that we can now track the activity of these key effector cells, which drive adverse left ventricular remodeling in patients. This provides the foundation and an opportunity for future research designed specifically to look at the effect of disease-modifying therapies^12^^,^^25^, ^26^, ^27^ on fibroblast activation and left ventricular remodeling after myocardial infarction. Our data have focused on myocardial infarction, but the same approach could in principle be used across all cardiomyopathic conditions.

The reasons underlying the accumulation of activated fibroblasts in the peri-infarct zone are not clear, although it has been previously observed in animal models of myocardial infarction as well as previous human ^68^Ga-FAPI PET studies.^6^^,^^9^^,^^17^ We suggest that the peri-infarct [^68^Ga]FAPI-46 signal could have several potential interpretations, including activated fibroblasts in transit to the infarct zone, proliferation of resident fibroblasts adjacent to the infarct, transient fibroblast activation in an area at risk salvaged by myocardial reperfusion, or activated fibroblasts with more of a regulatory role. Finally, our data demonstrating an association between segmental longitudinal strain and fibroblast activation suggest that myocardial deformation may act as a trigger to fibroblast activation in the peri-infarct zone, given the mechanotransducive properties of these cells.^28^

It could be argued that the differing resolutions of PET and magnetic resonance imaging might explain the differences in the signal. However, we believe that this is not the case, for the following reasons. First, we identified an altered T1 and T2 signal within the peri-infarct zone compared with the infarct and remote zones. This provides further independent evidence that this is a biologically active area where one might expect to see a difference in fibroblast activation. Second, the ratio between the volume of [^68^Ga]FAPI-46 and that of late gadolinium enhancement is highly dynamic over time. Indeed, the burden of [^68^Ga]FAPI-46 uptake was 183% greater than the burden of late gadolinium enhancement at week 1, with no difference by week 12. In the chronic infarcts, the [^68^Ga]FAPI-46 signal was more than 10-fold smaller than the region of late gadolinium enhancement. Any partial volume effect would be consistent across all scans, and so cannot explain the dynamic nature of our results. Third, we chose a strict threshold for defining increased [^68^Ga]FAPI-46 uptake at 1.5× the blood pool uptake for volumetric analysis while simultaneously adopting a generous approach to quantifying the myocardial infarct volume using the full-width half-maximum method for late gadolinium enhancement quantitation. Despite this, in the acute infarcts, the [^68^Ga]FAPI-46 signal extended well beyond the late gadolinium enhancement—nearly double in the early stages—a difference far greater than could be expected by a difference in resolution between the scan types. Fourth, when we analyzed for partial volume effects from the infarct zone into the left ventricular blood pool, increased activity was observed extending only 6.1 ± 3.6 mm in this radial direction. This was only one-third of the uptake that extended circumferentially beyond the region of late gadolinium enhancement into the surrounding peri-infarct zone of the myocardium. Finally, our histologic analysis confirmed the presence of FAP-positive fibroblasts in the infarct zone and peri-infarct zones of our patients who had sustained acute myocardial infarction. From an imaging perspective, the mismatch between the [^68^Ga]FAPI-46 uptake, late gadolinium enhancement, and T1 and T2 data confirms that these techniques provide different and complementary information, especially as the nature of the mismatch, particularly with late gadolinium enhancement, altered with time. Indeed, in chronic infarcts, the burden of [^68^Ga]FAPI-46 uptake was smaller than the infarct burden, being confined to the center of the infarct zone with no extension into the peri-infarct zone.

Although [^68^Ga]FAPI-46 uptake in the remote myocardium was lower than the peri-infarct and infarcted regions, the intensity of the signal was highly discriminative, with increased signal correlating with markers of left ventricular remodeling both during the acute phase and at 12 months. It is interesting that the 3 patients with the highest [^68^Ga]FAPI-46 uptake in the remote myocardium also had the most advanced left ventricular systolic dysfunction and adverse remodeling in our cohort. Our histologic findings also demonstrated intense fibroblast activation in the remote zone of patients with acute infarction, which is perhaps unsurprising given that the samples were obtained from patients who either died or required cardiac transplantation within 30 days of their acute infarct. These findings suggest that fibroblast activation in the remote myocardium may be most important in the most unwell patients thus, further studies are required to assess the role of activated fibroblasts in the remote myocardium of patients with heart failure and ischemic cardiomyopathy.

Our study demonstrated that fibroblast activation and fibrosis activity commonly extend beyond the left ventricle and into the right ventricle, which was observed in nearly two-thirds of patients with acute inferior myocardial infarction. Although this makes anatomical sense, given the common blood supply of the right ventricle and the inferior left ventricular wall, this proportion is much higher than previously reported with other imaging techniques. It highlights the sensitivity of [^68^Ga]FAPI-46 PET and illustrates its ability to detect fibrosis activity even in thin-walled myocardial structures, which previously have been difficult to identify with existing imaging approaches. This has implications for other right ventricular conditions such as arrhythmogenic ventricular cardiomyopathy or congenital heart diseases, where reliable fibrosis imaging is currently not possible.

The longitudinal design of our study with repeated cardiac magnetic resonance at 1 year after the acute infarct has allowed us to assess the relationship between baseline fibroblast activation and subsequent infarct size, cardiac remodeling, and left ventricular dysfunction. We have demonstrated that the degree of acute myocardial fibroblast activation is closely related to each of these markers of myocardial health 12 months later. Further work is required to investigate the precise nature of this relationship, but it does support the concept that activated fibroblasts play a key role in cardiac repair and remodeling after myocardial infarction and that they may well represent a promising target for therapeutic intervention. For the first time, the development of [^68^Ga]FAPI-46 PET now allows us to assess the effects of existing and future therapies on fibroblast activation, such as small molecular and mRNA-mediated antifibrotic agents, and whether modulation of these cells can have a favorable influence on cardiac repair and remodeling after myocardial infarction.^29^^,^^30^

### Study limitations

As for many studies investigating acute myocardial infarction, male participants constitute most of our clinical study population. Inevitably, we may have missed the most unwell patients after acute myocardial infarction who could not undergo the imaging protocol safely within the first week of their infarct. Subjects were predominantly white, reflecting the profile of our local population. Larger multicenter studies are therefore required to confirm the generalizability of our findings.

## Conclusions

Intense fibroblast activation peaks early after acute myocardial infarction and extends beyond the infarct zone into the peri-infarct area as well as the right ventricle and remote myocardium in some cases. Fibroblast activation remains high even after 3 months, with chronic fibroblast activity observed several years after the acute event. In the acute phase, the burden of fibroblast activation is nearly double the size of scar and is associated with infarct size, cardiac remodeling, and left ventricular systolic function at 1 year. These data suggest that activated fibroblasts play a key ongoing role in healing after myocardial infarction. [^68^Ga]FAPI-46 PET is able to track the activity of these key cells and thus may develop a role in evaluating the effects of existing and novel antifibrotic therapies.

## Funding Support and Author Disclosures

SOFIE provided the [^68^Ga] FAPI-46 precursor free of charge. They had no influence on the study design, its conduction, and collection and analysis of results, and although they have approved its content, they did not contribute to or influence the creation of this manuscript. There are no other declarations of relevance to this manuscript. Dr Barton is funded by the British Heart Foundation (SS/CH/09/002/26360). Dr Craig is funded by the Medical Research Council (MR/Y009932/1). Dr Loganath is funded by the British Heart Foundation (FS/CRTF/22/24377). Dr Joshi is funded by the British Heart Foundation (FS/CRTF/20/24087). Dr Tsampasian is supported by an NIHR Doctoral Fellowship Award (NIHR303306). Dr Tzolos is funded by the British Heart Foundation (FS/CRTF/20/24086). Dr Whittington is funded by the British Heart Foundation (FS/CRTF/21/24129). Dr Williams is funded by the British Heart Foundation (FS/ICRF/20/26002). Dr van Beek is supported by the Scottish Imaging Network: A Platform of Scientific Excellence. Dr Berman participates in software royalties for QPET software at Cedars-Sinai Medical Center; and is a consultant for GE Healthcare. Dr Dey is supported by the National Heart, Lung, and Blood Institute (1R01HL148787-01A1). Dr Slomka is supported by the National Heart, Lung, and Blood Institute (R35HL161195); participates in software royalties for QPET software at Cedars-Sinai Medical Center; and has received grants from Siemens Medical Systems. Dr Newby is funded by the British Heart Foundation (CH/09/002/26360, RG/F/22/110093, RE/24/130012). Dr Dweck is funded by the British Heart Foundation (FS/SCRF/21/32010). All other authors have reported that they have no relationships relevant to the contents of this paper to disclose.

## References

[1] Naghavi M., Libby P., Falk E. (2003). From vulnerable plaque to vulnerable patient: a call for new definitions and risk assessment strategies: part I. Circulation.

[2] Travers J., Kamal F., Robbins J., Yutzey K., Blaxall B.C. (2016). Cardiac fibrosis: the fibroblast awakens. Circ Res.

[3] Turner N.A. (2014). Effects of interleukin-1 on cardiac fibroblast function: relevance to post-myocardial infarction remodelling. Vascul Pharmacol.

[4] Porter K., Turner N.A. (2009). Cardiac fibroblasts: at the heart of myocardial remodeling. Pharmacol Ther.

[5] Jellis C., Martin J., Narula J., Marwick T.H. (2010). Assessment of nonischemic myocardial fibrosis. J Am Coll Cardiol.

[6] Varasteh Z., Mohanta S., Robu S. (2019). Molecular imaging of fibroblast activity after myocardial infarction using a (68)Ga-labeled fibroblast activation protein inhibitor, FAPI-04. J Nucl Med.

[7] Bing R., Dweck M. (2019). Myocardial fibrosis: why image, how to image and clinical implications. Heart.

[8] Everett R., Stirrat C., Semple S. (2016). Assessment of myocardial fibrosis with T1 mapping MRI. Clin Radiol.

[9] Nagaraju C., Dries E., Popovic N. (2017). Global fibroblast activation throughout the left ventricle but localized fibrosis after myocardial infarction. Sci Rep.

[10] Nagaraju C., Robinson E., Abdesselem M. (2019). Myofibroblast phenotype and reversibility of fibrosis in patients with end-stage heart failure. J Am Coll Cardiol.

[11] Tillmanns J., Hoffman D., Habbaba Y. (2015). Fibroblast activation protein alpha expression identifies activated fibroblasts after myocardial infarction. J Mol Cell Cardiol.

[12] Aghajanian H., Kimura T., Rurik J. (2019). Targeting cardiac fibrosis with engineered T cells. Nature.

[13] Barton A.K., Tzolos E., Bing R. (2023). Emerging molecular imaging targets and tools for myocardial fibrosis detection. Eur Heart J Cardiovasc Imaging.

[14] Notohamiprodjo S., Nekolla S., Robu S. (2022). Imaging of cardiac fibroblast activation in a patient after acute myocardial infarction using (68)Ga-FAPI-04. J Nucl Cardiol.

[15] Diekmann J., Koenig T., Thackeray J.T. (2022). Cardiac fibroblast activation in patients early after acute myocardial infarction: integration with magnetic resonance tissue characterization and subsequent functional outcome. J Nucl Med.

[16] Kessler L., Kupusovic J., Ferdinandus J. (2021). Visualization of fibroblast activation after myocardial infarction using 68Ga-FAPI PET. Clin Nucl Med.

[17] Diekmann J., Koenig T., Zwadlo C. (2021). Molecular imaging identifies fibroblast activation beyond the infarct region after acute myocardial infarction. J Am Coll Cardiol.

[18] Zhang M., Quan W., Zhu T. (2023). [(68)Ga]Ga-DOTA-FAPI-04 PET/MR in patients with acute myocardial infarction: potential role of predicting left ventricular remodeling. Eur J Nucl Med Mol Imaging.

[19] Siebermair J., Kohler M., Kupusovic J. (2021). Cardiac fibroblast activation detected by Ga-68 FAPI PET imaging as a potential novel biomarker of cardiac injury/remodeling. J Nucl Cardiol.

[20] Robson P.M., Dey D., Newby D.E. (2017). MR/PET imaging of the cardiovascular system. J Am Coll Cardiol Img.

[21] Nakazato R., Berman D.S., Dey D. (2012). Automated quantitative Rb-82 3D PET/CT myocardial perfusion imaging: normal limits and correlation with invasive coronary angiography. J Nucl Cardiol.

[22] Miller R.J.H., Cadet S., Pournazari P. (2022). Quantitative assessment of cardiac hypermetabolism and perfusion for diagnosis of cardiac sarcoidosis. J Nucl Cardiol.

[23] Cerqueira M.D., Weissman N.J., Dilsizian V. (2002). Standardized myocardial segmentation and nomenclature for tomographic imaging of the heart. A statement for healthcare professionals from the Cardiac Imaging Committee of the Council on Clinical Cardiology of the American Heart Association. Circulation.

[24] Altmann A., Haberkorn U., Siveke J. (2021). The latest developments in imaging of fibroblast activation protein. J Nucl Med.

[25] Lewis G.A., Dodd S., Clayton D. (2021). Pirfenidone in heart failure with preserved ejection fraction: a randomized phase 2 trial. Nat Med.

[26] Teerlink J.R., Cotter G., Davison B.A. (2013). Serelaxin, recombinant human relaxin-2, for treatment of acute heart failure (RELAX-AHF): a randomised, placebo-controlled trial. Lancet.

[27] Täubel J., Hauke W., Rump S. (2021). Novel antisense therapy targeting microRNA-132 in patients with heart failure: results of a first-in-human phase 1b randomized, double-blind, placebo-controlled study. Eur Heart J.

[28] Tian G., Ren T. (2023). Mechanical stress regulates the mechanotransduction and metabolism of cardiac fibroblasts in fibrotic cardiac diseases. Eur J Cell Biol.

[29] Ishikane S., Arioka M., Takahashi-Yanaga F. (2023). Promising small molecule anti-fibrotic agents: Newly developed or repositioned drugs targeting myofibroblast transdifferentiation. Biochem Pharmacol.

[30] Morfino P., Aimo A., Castiglione V., Gálvez-Montón C., Emdin M., Bayes-Genis A. (2023). Treatment of cardiac fibrosis: from neuro-hormonal inhibitors to CAR-T cell therapy. Heart Fail Rev.

