# Supplementary material for: Myocardial Fibroblast Activation After Acute Myocardial Infarction: A Positron Emission Tomography and Magnetic Resonance Study
Source: J Am Coll Cardiol. 2025 Feb 18;85(6):578–91. doi: 10.1016/j.jacc.2024.10.103 (PMC11835506; doi:10.1016/j.jacc.2024.10.103)
Supplement: Supplemental Material [file mmc1.docx]

**Myocardial Fibroblast Activation After Acute Myocardial Infarction:**

**A Positron Emission Tomography and Magnetic Resonance Study**

**SUPPLEMENTAL MATERIAL**

Anna K. Barton MD^a^, Neil Craig MD^a^, Krithika Loganath MD^a^, Shruti Joshi MD^a^, Vasiliki Tsampasian MD^b^, Menaka Mahendran MD^a^, Joel Lenell MD^c^, Evangelos Tzolos MD^a^, Trisha Singh MD^a,d^, Beth Whittington MD^a^, Jennifer Nash MD^a^, Michelle C Williams MBChB PhD^a^, Edwin J.R. van Beek MD PhD^e^, Mark G. MacAskill PhD^a^, Bronwyn Berkeley BSc^a^, Stefan Veizades MSc^a^, Mairi Brittan PhD^a^, Andrew H Baker PhD^a^, Stephanie Sellers PhD^f^, Alison Fletcher PhD^e^, Tim Clark BSc MSc Dip IPEM(S)^e^, Clint Waight BPharm MSc^g^, Riemer H. J. A. Slart MD^h^, Daniel Berman MD^i^, Damini Dey PhD^i^, Piotr Slomka PhD^i^, David E Newby MD PhD^a^, Marc R Dweck MD PhD^a^

1. Centre for Cardiovascular Science, The University of Edinburgh, 47-49 Little France Crescent, Edinburgh, Scotland, United Kingdom, EH16 4SB
2. Norwich Medical School, University of East Anglia, Norwich NR4 7UG
3. Uppsala Clinical Research Center, Uppsala University, Uppsala, Sweden
4. Department of Cardiology, Southampton General Hospital, University Hospital Southampton NHS Foundation Trust, Tremona Road, Southampton, Hampshire, SO16 6YD
5. Edinburgh Imaging Facility, Queen’s Medical Research Institute, 47 Little France Crescent, Edinburgh, Scotland, United Kingdom, EH16 4TJ
6. Department of Radiology and Centre for Heart Lung Innovation, St Paul’s Hospital and University of British Columbia, Vancouver, Canada
7. NHS Lothian, The Royal Infirmary of Edinburgh, 51 Little France Crescent, Edinburgh, Scotland, United Kingdom, EH16 4SA
8. Medical Imaging Center, Department of Nuclear Medicine & Molecular Imaging, University Medical Center Groningen, Groningen, The Netherlands
9. Departments of Medicine, Biomedical Sciences and Imaging, Cedars-Sinai Medical Centre, Los Angeles, California, 90048

**[^68^Ga]FAPI-46 Radiolabeling**

^68^Ga-labelling of FAPI-46 precursor (SOFIE, United States) was performed in the NHS Lothian Radiopharmacy Department in the Royal Infirmary of Edinburgh, under Good Manufacturing Practice conditions. Freeze-dried precursor (50 µg) was taken from -20 ^o^C storage and reconstituted with sterile water for injection, 3% sodium acetate solution, and 1% ascorbic acid solution before ^68^GaCl_3_ in 0.1 M hydrochloric acid was eluted from the ^68^Ga generator (IRE Elit), into the reaction vial. The reaction vial was then heated at 95.0 ^o^C for a pre-determined period of heating and cooling, before being diluted with water to its final volume.

**Positron Emission Tomography and Magnetic Resonance Imaging**

The magnetic resonance protocol included a free-breathing radial volumetric interpolated breath-hold examination sequence for attenuation correction, and long and short-axis stack cine imaging. The protocol also included late gadolinium enhancement images acquired in two full short-axis stacks in two phase-encoding directions 7-15 min post administration of 1 mg/kg gadobutrol (Gadovist, Bayer Inc., Missisauga, Canada). Contrast imaging was not performed at weeks 2 and 4 in those patients undergoing serial magnetic resonance scans to limit contrast medium exposure. Native T1 and T2 mapping sequences were acquired in three short axis views of the left ventricle at the base, mid-cavity and apical levels.

Positron emission tomography images were reconstructed using an Ordered Subset Expectation Maximisation (OSEM) iterative algorithm, undergoing 3 iterations of 21 subsets. Reconstructions were scaled to a 344 x 344 pixel matrix with a zoom factor of 1, giving voxel dimensions of 2.09 x 2.09 x 2.03 mm. Gaussian smoothing was applied with a 5-mm Full-Width at Half-Maximum kernel. New York reconstructions included an otherwise equivalent OSEM reconstruction featuring point spread function modeling and were used to quantify [^68^Ga]FAPI-46 signal.

**Histological Analysis**

Formalin-fixed paraffin-embedded human heart tissue was cut into 4-60°C. Prior to staining, slides were dewaxed through three changes of xylene (10467270, Fisher Scientific), for 5 minutes each, then rehydrated by three changes of absolute ethanol (12498740, Fisher Scientific) and three changes of ethanol increasingly diluted with distilled water (95%, 80% and 70% ethanol) for 20 seconds each.

For staining of fibrosis, slides underwent trichrome staining (ab150686, abcam), according to the reagent manufacturer instructions.

For detection of FAP, slides underwent immunohistochemistry with a FAP (ab207178, abcam) antibody (1:400 dilution). Prior to staining, slides were incubated in Tris-EDTA antigen retrieval buffer (15576276, Fisher Scientific) in a 60°C oven overnight. Immunohistochemical staining was performed using the Leica Bond RX Fully Automated Research Stainer and the Bond Polymer Refine Detection Kit (DS9800, Leica Biosystems), according to the standard manufacturer protocol.

Once stained, slides were dehydrated by two changes of absolute ethanol and cleared by two changes of xylene, each for 5 minutes. Slides were then coverslipped with Pertex mounting medium (SEA-0100-00A, CellPath). Sections were imaged with AxioScan Z1 slide scanner at 20x magnification.

| Supplemental Table 1 – Global Gallium-68-Fibroblast Activation Protein Inhibitor-46 Positron Emission Tomography and Magnetic Resonance Analysis | | | | | | | | |
| --- | --- | --- | --- | --- | --- | --- | --- | --- |
|  | **Acute**  **myocardial**  **infarction** | | | | p values  for trends across weeks 1,2,4,12 | **Chronic**  **myocardial infarction** | **Control**  **volunteers** | p values  Chronic myocardial infarction versus Control Volunteers |
| Timepoint post-infarct | **Week 1** | **Week 2** | **Week 4** | **Week 12** | **-** | **>24 months** | **-** | **-** |
| Time since infarct | 8±2 days | 15±2 days | 32±10 days | 88±9 days | - | 7±6 years | - | - |
|  | **Magnetic resonance imaging** | | | | | | | |
| Left ventricular ejection fraction (%) | 58±8 | 57±9 | 56±9 | 60±6 | p=0.60 | 59±8 | 67±5 | **p<0.01** |
| Indexed left ventricular end diastolic volume (mL/m^2^) | 79±14 | 79±14 | 81±21 | 78±15 | p=0.80 | 75±14 | 73±12 | p=0.60 |
| Infarct volume (mL) | 25.5±18.1 | - | - | 16.1±8.3 | p=0.06 | 10.6±8.0 | 0 | **p<0.01** |
| Infarct burden (%LGE) | 15.2±10.6 | - | - | 10.3±6.1 | p=0.07 | 8.1±7.2 | 0 | **p<0.01** |
|  | **[^68^Ga]FAPI-46 Positron emission tomography** | | | | | | | |
| Injected [^68^Ga]FAPI-46 dose (mBq/kq) | 1.4±0.5 | 1.3±0.3 | 1.3±0.4 | 1.3±0.4 | p=0.98 | 1.4±0.4 | 1.3±0.4 | p=0.15 |
| Right atrial bloodpool SUV_mean_ | 1.3±0.2 | 1.3±0.2 | 1.4±0.2 | 1.4±0.4 | p=0.33 | 1.3±0.3 | 1.3±0.3 | p=0.62 |
| Visually positive myocardial [^68^Ga]FAPI-46 | 27 (100%) | 30 (100%) | 21 (100%) | 17 (89%) | **-** | 14 (70%) | 0 (0%) | **-** |
| Myocardial SUV_max_ | 5.3±1.5 | 4.8±1.3 | 4.2±1.3 | 3.8±1.2 | **p=0.001** | 2.1±0.6 | 1.5±0.3 | **p<0.001** |
| Myocardial TBR_max_ | 4.0±1.1 | 3.7±1.0 | 3.1±0.8 | 2.7±0.7 | **p<0.001** | 1.7±0.5 | 1.2±0.1 | **p<0.001** |
| Myocardial SUV_mean_ | 2.0±0.5 | 1.9±0.4 | 1.8±0.5 | 1.7±0.5 | **p<0.05** | 1.2±0.2 | 1.0±0.2 | **p<0.01** |
| Myocardial TBR_mean_ | 1.5±0.3 | 1.4±0.3 | 1.3±0.3 | 1.2±0.2 | **p<0.01** | 0.9±0.1 | 0.8±0.1 | **p<0.001** |
| Volume of fibroblast activation (mL) | 49.5±23.6 | 43.3±30.5 | 39.7±39.5 | 24.1±15.9 | **p<0.01** | 0.9±3.3 | 0 | **p<0.05** |
| Burden of fibroblast activation  (% myocardium with increased [^68^Ga]FAPI-46) | 27.8±12.4 | 23.0±14.4 | 19.6±14.6 | 12.8±8.1 | **p<0.01** | 0.6±2.2 | 0 | p=0.26 |

LGE = late gadolinium enhancement, [^68^Ga]FAPI-46 = gallium-68 fibroblast activation protein inhibitor-46, SUV_mean_ = mean standardized uptake value, SUV_max_ = maximum standardized uptake value, TBR_max_ = maximum target to background ratio, TBR_mean_ = mean target to background ratio.

| \| Supplemental Table 2 – Regional Gallium-68-Fibroblast Activation Protein Inhibitor-46 Positron Emission Tomography and Magnetic Resonance Analysis \| \| --- \| | | | | | |
| --- | --- | --- | --- | --- | --- | --- |
| Timepoint post-myocardial infarction | **Acute myocardial infarction**  **Weeks 1-4** | **Acute myocardial infarction**  **Week 12** | p values  Weeks 1-4 versus Week 12 | **Chronic myocardial infarction**  **>24 months** | p values  Week 12 versus Chronic myocardial infarction |
| Time since infarct | 11±5 days | 88±9 days | - | 7.1±6 years | - |
| n | 40 | 19 | - | 20 | - |
| **Magnetic resonance imaging** | | | | | |
| Left Ventricular Ejection Fraction (%) | 57±10 | 60±6 | p=0.30 | 59±8 | p=0.96 |
| Indexed left ventricular end- diastolic volume (mL/m^2^) | 82±18 | 78±15 | p=0.22 | 75±14 | p=0.81 |
| Infarct burden (%) | 14.5±11.4 | 11.5±5.2 | p=0.13 | 8.1±7.2 | p=0.32 |
| Presence of right ventricular late gadolinium enhancement | 4 (10%) | 4 (21%) | - | 0 (0%) | - |
| **[^68^Ga]FAPI-46 positron emission tomography** | | | | | |
| **Infarct Zone** | | | | | |
| Visually increased [^68^Ga]FAPI-46 activity in infarct zone | 40 (100%) | 17 (89%) | - | 14 (70%) | - |
| Infarct SUV_max_* | 5.5±1.5 | 4.7±1.5 | p=0.06 | 2.5±0.6 | **p=0.001** |
| Infarct TBR_max_* | 4.1±1.1 | 3.0±0.7 | **p<0.001** | 1.9±0.5 | **p=0.001** |
| **Peri-infarct Zone** | | | | | |
| Visually increased [^68^Ga]FAPI-46 activity in peri-infarct zone | 37 (95%) | 15 (79%) | - | 1 (5%) | - |
| Volume of peri-infarct [^68^Ga]FAPI-46 uptake (mL)* | 28.9±21.8 | 10.0±8.6 | **p<0.001** | 8.3 | ** |
| Burden of peri-infarct [^68^Ga]FAPI-46 uptake (%)* | 12.1±8.7 | 2.8±4.0 | **p<0.001** | 3.5 | ** |
| Peri-infarct SUV_max_* | 4.7±1.3 | 4.2±1.4 | p=0.20 | 2.8 | ** |
| Peri-infarct TBR_max_* | 3.3±0.8 | 2.7±0.4 | **p=0.01** | 2.5 | ** |
| **Remote Myocardium** | | | | | |
| Visually increased [^68^Ga]FAPI-46 activity in remote myocardium | 1 (2.5%) | 0 (0%) | - | 0 (0%) | - |
| Remote myocardium SUV_max_ | 1.4±0.3 | 1.5±0.5 | p=0.15 | 1.3±0.3 | p=0.17 |
| Remote myocardium TBR_max_ | 1.0±0.1 | 1.0±0.1 | p=0.52 | 1.0±0.2 | p=0.98 |
| **Right Ventricle** | | | | | |
| Visually increased [^68^Ga]FAPI-46 uptake in right ventricle | 15 (37.5%) | 2 (10.5%) | - | 0 (0%) | - |
| Right Ventricular SUV_max_* | 3.0±0.6 | 2.5±0.1 | p=0.31 | n/a | - |
| Right Ventricular TBR_max_* | 2.2±0.5 | 2.0±0.1 | p=0.43 | n/a | - |

LGE = late gadolinium enhancement, [^68^Ga]FAPI-46 = gallium-68 fibroblast activation protein inhibitor-46, SUV_max_ = maximum standardized uptake value, TBR_max_ = maximum target to background ratio

* Data presented only from participants with visually increased [^68^Ga]FAPI-46 uptake

** Statistical comparisons not performed as n=1 for chronic MI group

| **Table 3a: Anterior Infarcts** | | | | | | | | | | | | | |
| --- | --- | --- | --- | --- | --- | --- | --- | --- | --- | --- | --- | --- | --- |
|  | | SUVmax | | | | | | | SUVmean | | | | |
| AHA Segment | | Week 1  (n=10) | | Week 2  (n=8) | Week 4  (n=6) | Week 12  (n=6) | | p value | Week 1  (n=10) | Week 2  (n=8) | Week 4  (n=6) | Week 12  (n=6) | p value |
| 1 | | 2.9±1.3 | | 2.8±1.5 | 2.8±1.8 | 1.8±0.9 | | p=0.44 | 1.8±0.8 | 1.9±1.0 | 2.0±0.5 | 1.2±0.6 | p=0.58 |
| 2 | | 3.1±1.5 | | 3.3±1.5 | 2.8±1.4 | 1.9±1.0 | | p=0.32 | 2.1±0.9 | 2.3±1.1 | 2.1±1.1 | 1.5±0.7 | p=0.57 |
| 3 | | 2.5±1.1 | | 2.6±1.2 | 2.3±0.8 | 1.9±0.6 | | p=0.62 | 1.8±0.7 | 1.7±0.6 | 1.6±0.4 | 1.5±0.4 | p=0.81 |
| 4 | | 1.8±0.4 | | 1.9±0.5 | 1.9±1.3 | 1.8±0.4 | | p=0.97 | 1.5±0.3 | 1.4±0.3 | 1.4±0.4 | 1.4±0.3 | p=0.89 |
| 5 | | 1.6±0.5 | | 1.5±0.2 | 2.3±1.9 | 1.6±0.5 | | p=0.74 | 1.2±0.4 | 1.1±0.1 | 1.4±1.0 | 1.2±0.4 | p=0.84 |
| 6 | | 2.0±0.9 | | 2.0±1.2 | 2.3±1.9 | 1.6±0.6 | | p=0.79 | 1.3±0.4 | 1.3±0.4 | 1.6±1.4 | 1.1±0.4 | p=0.62 |
| 7 | | 4.2±1.1 | | 4.0±1.1 | 3.6±1.7 | 2.6±1.1 | | p=0.08 | 2.7±1.3 | 2.7±1.1 | 2.6±1.5 | 1.7±0.9 | p=0.37 |
| 8 | | 4.9±0.9 | | 4.7±1.0 | 4.1±1.0 | 3.3±1.1 | | **p<0.05** | 3.5±1.1 | 3.4±0.9 | 2.9±1.0 | 2.2±1.0 | p=0.08 |
| 9 | | 4.2±1.2 | | 4.1±0.8 | 3.3±0.8 | 2.9±1.0 | | p=0.05 | 2.5±0.9 | 2.4±0.8 | 1.9±0.6 | 1.7±0.6 | p=0.19 |
| 10 | | 2.0±0.4 | | 2.0±0.7 | 1.9±0.7 | 1.9±0.3 | | p=0.87 | 1.5±0.4 | 1.5±0.5 | 1.4±0.4 | 1.4±0.3 | p=0.98 |
| 11 | | 1.7±0.7 | | 1.5±0.3 | 1.9±1.3 | 1.6±0.5 | | p=0.84 | 1.2±0.4 | 1.2±0.2 | 1.4±1.0 | 1.2±0.3 | p=0.84 |
| 12 | | 2.8±1.5 | | 2.6±1.3 | 2.8±1.8 | 1.9±0.8 | | p=0.65 | 1.7±0.8 | 1.6±0.6 | 1.9±1.3 | 1.3±0.5 | p=0.65 |
| 13 | | 5.7±1.5 | | 5.1±1.2 | 4.3±1.3 | 3.4±0.8 | | **p<0.05** | 3.6±1.0 | 3.5±1.1 | 3.1±1.3 | 2.2±0.8 | p=0.11 |
| 14 | | 5.5±1.2 | | 5.2±1.2 | 4.3±0.8 | 3.7±1.2 | | **p<0.05** | 4.1±0.9 | 3.9±0.6 | 3.2±0.7 | 2.7±0.9 | **p<0.05** |
| 15 | | 4.1±1.0 | | 3.8±0.9 | 3.0±0.6 | 2.9±0.8 | | **p<0.05** | 2.2±0.5 | 2.2±0.6 | 1.8±0.4 | 1.7±0.4 | p=0.13 |
| 16 | | 3.7±1.6 | | 3.9±1.5 | 3.0±1.5 | 2.4±0.9 | | p=0.19 | 2.1±1.0 | 2.0±0.6 | 1.9±1.0 | 1.5±0.5 | p=0.57 |
| 17 | | 5.9±1.6 | | 5.2±1.0 | 4.3±1.1 | 3.7±1.2 | | **p<0.05** | 3.8±0.9 | 3.6±0.9 | 2.9±0.9 | 2.6±1.0 | p=0.07 |
|  | | | | | | | | | | | | | |
| **Table 3b: Inferior Infarcts** | | | | | | | | | | | | | |
|  | SUVmax | | | | | | | | SUVmean | | | | |
| AHA Segment | Week 1  (n=14) | | Week 2  (n=20) | | Week 4  (n=13) | Week 12  (n=12) | | p value | Week 1  (n=14) | Week 2  (n=20) | Week 4  (n=13) | Week 12  (n=12) | p value |
| 1 | 1.6±0.3 | | 1.5±0.2 | | 1.5±0.3 | 1.7±0.5 | | p=0.52 | 1.2±0.2 | 1.2±0.2 | 1.2±0.2 | 1.3±0.3 | p=0.33 |
| 2 | 1.9±0.4 | | 1.7±0.3 | | 1.7±0.3 | 1.9±0.6 | | p=0.20 | 1.4±0.2 | 1.3±0.2 | 1.3±0.2 | 1.5±0.4 | p=0.39 |
| 3 | 3.6±1.3 | | 3.3±1.1 | | 2.9±1.0 | 2.9±0.9 | | p=0.29 | 2.4±0.7 | 2.1±0.6 | 1.8±0.5 | 2.0±0.6 | p=0.14 |
| 4 | 3.9±1.3 | | 3.7±1.2 | | 3.2±1.2 | 3.1±1.0 | | p=0.23 | 2.8±1.0 | 2.5±0.8 | 2.2±0.8 | 2.2±0.8 | p=0.22 |
| 5 | 2.7±1.0 | | 2.6±1.1 | | 2.3±1.0 | 2.4±0.8 | | p=0.73 | 1.6±0.5 | 1.4±0.4 | 1.4±0.4 | 1.5±0.4 | p=0.61 |
| 6 | 1.6±0.4 | | 1.5±0.4 | | 1.5±0.3 | 1.7±0.5 | | p=0.48 | 1.2±0.3 | 1.1±0.2 | 1.1±0.3 | 1.3±0.3 | p=0.23 |
| 7 | 1.4±0.2 | | 1.4±0.3 | | 1.4±0.3 | 1.5±0.4 | | p=0.50 | 1.1±0.2 | 1.1±0.2 | 1.1±0.2 | 1.2±0.3 | p=0.33 |
| 8 | 1.6±0.3 | | 1.5±0.3 | | 1.5±0.3 | 1.6±0.5 | | p=0.85 | 1.2±0.2 | 1.2±0.2 | 1.2±0.2 | 1.3±0.3 | p=0.59 |
| 9 | 4.1±1.4 | | 3.6±1.4 | | 3.2±1.1 | 2.9±1.0 | | p=0.10 | 2.4±0.8 | 2.1±0.7 | 1.8±9.5 | 1.9±0.5 | p=0.10 |
| 10 | 4.5±1.4 | | 4.2±1.3 | | 3.7±1.2 | 3.3±1.0 | | p=0.09 | 3.3±1.0 | 3.2±0.2 | 2.7±0.9 | 2.6±0.8 | p=0.24 |
| 11 | 3.2±1.5 | | 3.1±1.3 | | 2.6±1.2 | 2.5±0.8 | | p=0.34 | 1.9±0.8 | 1.8±0.8 | 1.7±0.8 | 1.6±0.4 | p=0.79 |
| 12 | 1.6±0.4 | | 1.4±0.4 | | 1.5±0.3 | 1.6±0.3 | | p=0.52 | 1.2±0.2 | 1.1±0.2 | 1.7±2.2 | 1.2±0.2 | p=0.32 |
| 13 | 1.4±0.3 | | 1.4±0.4 | | 1.4±0.3 | 1.5±0.4 | | p=0.72 | 1.1±0.2 | 1.1±0.2 | 1.1±0.2 | 1.2±0.3 | p=0.35 |
| 14 | 3.1±1.3 | | 2.5±0.9 | | 2.1±0.6 | 2.3±1.1 | | p=0.07 | 1.5±0.3 | 1.4±0.3 | 1.3±0.3 | 1.5±0.5 | p=0.34 |
| 15 | 4.4±1.6 | | 3.9±1.6 | | 3.6±1.5 | 3.4±1.3 | | p=0.37 | 3.1±1.1 | 2.7±1.1 | 2.5±0.9 | 2.3±0.9 | p=0.21 |
| 16 | 3.0±1.3 | | 2.6±1.3 | | 2.4±1.1 | 2.2±0.6 | | p=0.32 | 1.7±0.7 | 1.5±0.6 | 1.4±0.6 | 1.4±0.2 | p=0.57 |
| 17 | 3.2±1.4 | | 2.5±1.4 | | 2.4±1.2 | 2.5±1.4 | | p=0.41 | 1.6±0.5 | 1.9±1.8 | 1.4±0.5 | 1.5±1.5 | p=0.67 |
| **Table 3c: Lateral Infarcts** | | | | | | | | | | | | | |
|  | SUVmax | | | | | | | | SUVmean | | | | |
| AHA Segment | Week 1  (n=3) | | Week 2  (n=2) | | Week 4  (n=2) | Week 12  (n=1) | p value | | Week 1 | Week 2 | Week 4  (n=2) | Week 12  (n=1) | p value |
| 1 | 2.3±0.9 | | 2.2±0.1 | | 2.9±1.3 | 1.7 | * | | 1.5±0.6 | 1.5±0.1 | 2.2±0.8 | 1.4 | * |
| 2 | 1.7±0.3 | | 1.8±0.1 | | 2.5±0.8 | 1.7 | * | | 1.4±0.2 | 1.5±0.1 | 1.9±0.6 | 1.4 | * |
| 3 | 1.7±0.2 | | 1.9±0.0 | | 2.0±0.1 | 1.7 | * | | 1.4±0.2 | 1.6±0.2 | 1.7±0.1 | 1.5 | * |
| 4 | 2.5±1.7 | | 2.4±0.8 | | 2.6±0.8 | 1.8 | * | | 1.5±0.6 | 1.6±0.0 | 1.9±0.2 | 1.4 | * |
| 5 | 3.0±1.8 | | 3.3±0.1 | | 3.2±0.3 | 2.6 | * | | 1.8±0.7 | 1.9±0.1 | 2.1±0.4 | 1.8 | * |
| 6 | 2.7±1.3 | | 2.7±0.5 | | 2.9±0.0 | 2.2 | * | | 1.6±0.6 | 1.6±0.4 | 2.0±0.6 | 1.8 | * |
| 7 | 2.6±0.7 | | 2.1±0.4 | | 3.1±1.1 | 1.8 | * | | 1.8±0.5 | 1.5±0.3 | 2.2±1.0 | 1.3 | * |
| 8 | 2.3±1.5 | | 1.6±0.1 | | 2.8±1.5 | 1.4 | * | | 1.4±0.4 | 1.3±0.1 | 2.0±1.0 | 1.2 | * |
| 9 | 1.7±0.2 | | 1.8±0.1 | | 2.0±0.4 | 1.9 | * | | 1.2±0.0 | 1.3±0.1 | 1.5±0.1 | 1.4 | * |
| 10 | 3.5±2.0 | | 3.9±0.7 | | 3.9±0.9 | 3.7 | * | | 1.9±0.8 | 2.2±0.4 | 2.1±0.2 | 2.1 | * |
| 11 | 4.2±1.7 | | 4.3±0.2 | | 4.1±0.0 | 3.3 | * | | 2.9±1.9 | 3.0±1.6 | 2.9±1.5 | 2.4 | * |
| 12 | 3.8±1.0 | | 3.6±0.1 | | 3.2±0.3 | 2.8 | * | | 2.7±0.8 | 2.5±0.1 | 2.5±0.2 | 2.1 | * |
| 13 | 3.9±0.8 | | 2.6±0.4 | | 3.2±1.0 | 2.1 | * | | 2.2±0.8 | 1.5±0.2 | 2.2±1.1 | 1.3 | * |
| 14 | 2.3±1.1 | | 2.6±0.4 | | 2.9±1.7 | 1.6 | * | | 1.4±0.4 | 1.3±0.1 | 2.2±1.1 | 1.3 | * |
| 15 | 4.4±2.0 | | 5.1±0.1 | | 4.6±0.1 | 4.1 | * | | 2.6±1.1 | 2.9±0.3 | 2.8±2.1 | 2.7 | * |
| 16 | 5.3±1.3 | | 5.0±0.5 | | 4.2±0.1 | 3.9 | * | | 3.7±1.0 | 3.5±0.6 | 3.2±0.1 | 2.5 | * |
| 17 | 5.5±0.5 | | 5.0±0.3 | | 4.1±0.4 | 3.6 | * | | 2.7±1.4 | 2.3±0.4 | 2.4±0.6 | 1.8 | * |
|  | | | | | | | | | | | | | |
| **Table 3d: Anterior Infarcts** | | | | | | | | | | | | | |
|  | TBRmax | | | | | | | | | | | | |
| AHA Segment | Week 1  (n=10) | | | | Week 2  (n=8) | | | Week 4  (n=6) | | Week 12  (n=6) | | p value | |
| 1 | 1.9±0.7 | | | | 2.1±1.1 | | | 1.8±0.7 | | 1.2±0.5 | | p=0.22 | |
| 2 | 2.1±0.7 | | | | 2.4±1.1 | | | 1.9±0.8 | | 1.3±0.5 | | p=0.12 | |
| 3 | 1.7±0.6 | | | | 1.9±0.7 | | | 1.6±0.5 | | 1.3±0.3 | | p=0.32 | |
| 4 | 1.3±0.1 | | | | 1.4±0.3 | | | 1.3±0.2 | | 1.2±0.1 | | p=0.20 | |
| 5 | 1.1±0.2 | | | | 1.1±0.2 | | | 1.2±0.5 | | 1.1±0.1 | | p=0.80 | |
| 6 | 1.4±0.6 | | | | 1.5±1.0 | | | 1.5±0.8 | | 1.1±0.3 | | p=0.73 | |
| 7 | 2.9±0.6 | | | | 3.0±0.8 | | | 2.4±0.8 | | 1.7±0.6 | | **p<0.05** | |
| 8 | 3.4±0.6 | | | | 3.6±0.8 | | | 2.8±0.4 | | 2.2±0.5 | | **p<0.05** | |
| 9 | 3.0±0.9 | | | | 3.1±0.7 | | | 2.3±0.6 | | 2.0±0.5 | | **p<0.05** | |
| 10 | 1.4±0.2 | | | | 1.5±0.4 | | | 1.3±0.2 | | 1.3±0.3 | | p=0.39 | |
| 11 | 1.2±0.2 | | | | 1.2±0.2 | | | 1.2±0.5 | | 1.1±0.1 | | p=0.88 | |
| 12 | 1.9±0.8 | | | | 1.9±1.0 | | | 1.8±0.8 | | 1.3±0.5 | | p=0.55 | |
| 13 | 4.0±1.0 | | | | 3.9±.1 | | | 3.0±0.5 | | 2.4±0.4 | | **p<0.001** | |
| 14 | 3.8±0.9 | | | | 4.0±0.9 | | | 2.1±0.5 | | 2.6±0.3 | | **p<0.05** | |
| 15 | 2.9±07 | | | | 2.8±0.7 | | | 2.1±0.5 | | 2.1±0.5 | | **p<0.05** | |
| 16 | 2.5±0.8 | | | | 3.0±1.1 | | | 2.0±0.8 | | 1.7±0.7 | | p=0.07 | |
| 17 | 4.1±1.1 | | | | 4.0±1.0 | | | 3.0±0.7 | | 2.6±0.5 | | **p<0.05** | |
|  | | | | | | | | | | | | | |
| **Table 3e: Inferior Infarcts** | | | | | | | | | | | | | |
|  | TBRmax | | | | | | | | | | | | |
| AHA Segment | Week 1  (n=14) | | | | Week 2  (n=20) | | | Week 4  (n=13) | | Week 12  (n=12) | | p value | |
| 1 | 1.2±0.2 | | | | 1.2±0.2 | | | 1.2±0.2 | | 1.2±0.1 | | p=0.82 | |
| 2 | 1.5±0.3 | | | | 1.3±0.3 | | | 1.3±0.2 | | 1.3±0.2 | | p=0.18 | |
| 3 | 2.8±0.9 | | | | 2.6±1.0 | | | 2.2±0.8 | | 2.1±0.6 | | p=0.15 | |
| 4 | 3.0±1.0 | | | | 3.0±1.0 | | | 2.5±0.9 | | 2.2±0.7 | | p=0.07 | |
| 5 | 2.1±0.8 | | | | 2.1±0.8 | | | 1.8±0.7 | | 1.7±0.6 | | p=0.40 | |
| 6 | 1.2±0.3 | | | | 1.2±0.3 | | | 1.1±0.2 | | 1.2±0.2 | | p=0.76 | |
| 7 | 1.1±0.1 | | | | 1.1±0.1 | | | 1.1±0.1 | | 1.1±0.1 | | p=1.00 | |
| 8 | 1.2±0.0 | | | | 1.2±0.3 | | | 1.2±0.2 | | 1.1±0.1 | | p=0.62 | |
| 9 | 3.1±1.0 | | | | 2.9±1.3 | | | 2.4±0.8 | | 2.1±0.6 | | **p<0.05** | |
| 10 | 3.4±0.9 | | | | 3.4±1.0 | | | 2.8±0.8 | | 2.4±0.8 | | **p<0.05** | |
| 11 | 2.5±1.2 | | | | 2.5±1.1 | | | 2.0±0.9 | | 1.9±0.8 | | p=0.24 | |
| 12 | 1.3±0.4 | | | | 1.1±0.3 | | | 1.1±0.3 | | 1.1±0.2 | | p=0.37 | |
| 13 | 1.1±0.2 | | | | 1.1±0.2 | | | 1.1±0.1 | | 1.1±0.1 | | p=0.93 | |
| 14 | 2.4±1.0 | | | | 2.1±1.0 | | | 1.6±0.4 | | 1.6±0.7 | | **p<0.05** | |
| 15 | 3.4±1.1 | | | | 3.1±1.3 | | | 2.8±1.0 | | 2.5±0.9 | | p=0.17 | |
| 16 | 2.3±1.1 | | | | 2.1±1.0 | | | 1.8±0.8 | | 1.7±0.7 | | p=0.26 | |
| 17 | 2.5±1.1 | | | | 2.0±1.1 | | | 1.8±0.8 | | 1.8±0.7 | | p=0.26 | |
|  |  | | | |  | | |  | |  | |  | |
| **Table 3f: Lateral Infarcts** | | | | | | | | | | | | | |
|  | TBRmax | | | | | | | | | | | | |
| AHA Segment | Week 1  (n=3) | | | | Week 2  (n=2) | | | Week 4  (n=2) | | Week 12  (n=1) | | p value | |
| 1 | 2.0±0.9 | | | | 1.6±0.2 | | | 2.2±1.1 | | 1.3 | | * | |
| 2 | 1.4±0.2 | | | | 1.3±0.0 | | | 1.9±0.7 | | 1.3 | | * | |
| 3 | 1.4±0.2 | | | | 1.4±0.1 | | | 1.4±0.1 | | 1.3 | | * | |
| 4 | 2.2±1.6 | | | | 1.8±0.7 | | | 1.9±0.7 | | 1.4 | | * | |
| 5 | 2.6±1.7 | | | | 2.5±0.2 | | | 2.4±0.1 | | 2.0 | | * | |
| 6 | 2.3±1.2 | | | | 2.0±0.3 | | | 2.2±0.1 | | 1.7 | | * | |
| 7 | 2.2±0.6 | | | | 1.5±0.3 | | | 2.3±0.9 | | 1.4 | | * | |
| 8 | 1.9±1.2 | | | | 1.2±0.0 | | | 2.1±1.2 | | 1.1 | | * | |
| 9 | 1.4±0.1 | | | | 1.3±0.0 | | | 1.5±0.4 | | 1.5 | | * | |
| 10 | 3.0±1.8 | | | | 2.9±0.4 | | | 2.8±0.5 | | 2.8 | | * | |
| 11 | 3.6±1.7 | | | | 3.2±0.3 | | | 3.0±0.2 | | 2.5 | | * | |
| 12 | 3.2±1.2 | | | | 2.6±0.2 | | | 2.4±0.1 | | 2.2 | | * | |
| 13 | 3.2±0.5 | | | | 1.9±0.4 | | | 2.4±0.9 | | 1.6 | | * | |
| 14 | 1.9±0.9 | | | | 1.1±0.0 | | | 2.2±1.4 | | 1.2 | | * | |
| 15 | 3.7±1.9 | | | | 3.7±0.1 | | | 3.4±0.3 | | 3.2 | | * | |
| 16 | 4.5±1.4 | | | | 3.7±0.6 | | | 3.1±0.1 | | 3.0 | | * | |
| 17 | 4.7±0.8 | | | | 3.7±0.4 | | | 3.0±0.2 | | 2.8 | | * | |

**Supplemental Tables 3a-3f:** Segmental [^68^Ga]FAPI-46 analysis as per the AHA 17 segment myocardial model. Tables 4a-4c report the SUVmax and SUVmean signal over time. Table 4d demonstrates the segmental change in TBRmax over time in the anterior infarcts. Statistically significant change in an individual segment over time is presented in bold. *Statistical analysis not performed due to n=1 at Week 12

| Timepoint post myocardial infarction | Baseline  (Weeks 1-4) | Follow Up  52 weeks | p values  Weeks 1-4 versus Week 52 |
| --- | --- | --- | --- |
| Time since infarct (days) | 11±5 | 381±25 | - |
| n | 35 | 35 | - |
|  | **Magnetic resonance imaging** | | |
| Left Ventricular Ejection Fraction (%) | 57±11 | 58±9 | p=0.08 |
| Indexed left ventricular end diastolic volume (mL/m^2^) | 82±19 | 78±15 | p<0.05 |
| Infarct volume (mL)* | 25.0±21.0 | 14.9±12.9 | p<0.001 |
| Infarct burden (%)* | 15.4±12.0 | 10.5±7.5 | p<0.001 |
|  | **[^68^Ga]FAPI-46 Positron emission tomography** | | |
| Volume of fibroblast activation (mL) | 175.3±122.5 | - | - |
| Burden of fibroblast activation  (% myocardium with increased [^68^Ga]FAPI-46) | 26.1±15.9 | - | - |
| Infarct [^68^Ga]FAPI-46 TBR_max_ | 3.8±1.1 | - | - |
| Infarct [^68^Ga]FAPI-46 SUV_max_ | 5.1±1.6 | - | - |

**Supplemental** **Table 4: Baseline and follow up imaging results for acute MI participants undergoing 12-month follow up**

[^68^Ga]FAPI-46 = gallium-68 fibroblast activation protein inhibitor, SUV_max_ = maximum standardized uptake value, TBR_max_ = maximum target to background ratio

* Not including the 2 participants without interpretable post gadolinium contrast imaging

**Supplemental Figure 1. Study flow chart.**

The different study groups and their respective imaging procedures are outlined.

[^68^Ga]FAPI-46 = ^68^Gallium-Fibroblast Activation Protein Inhibitor-46, PET = Positron Emission Tomography, MR = Magnetic Resonance


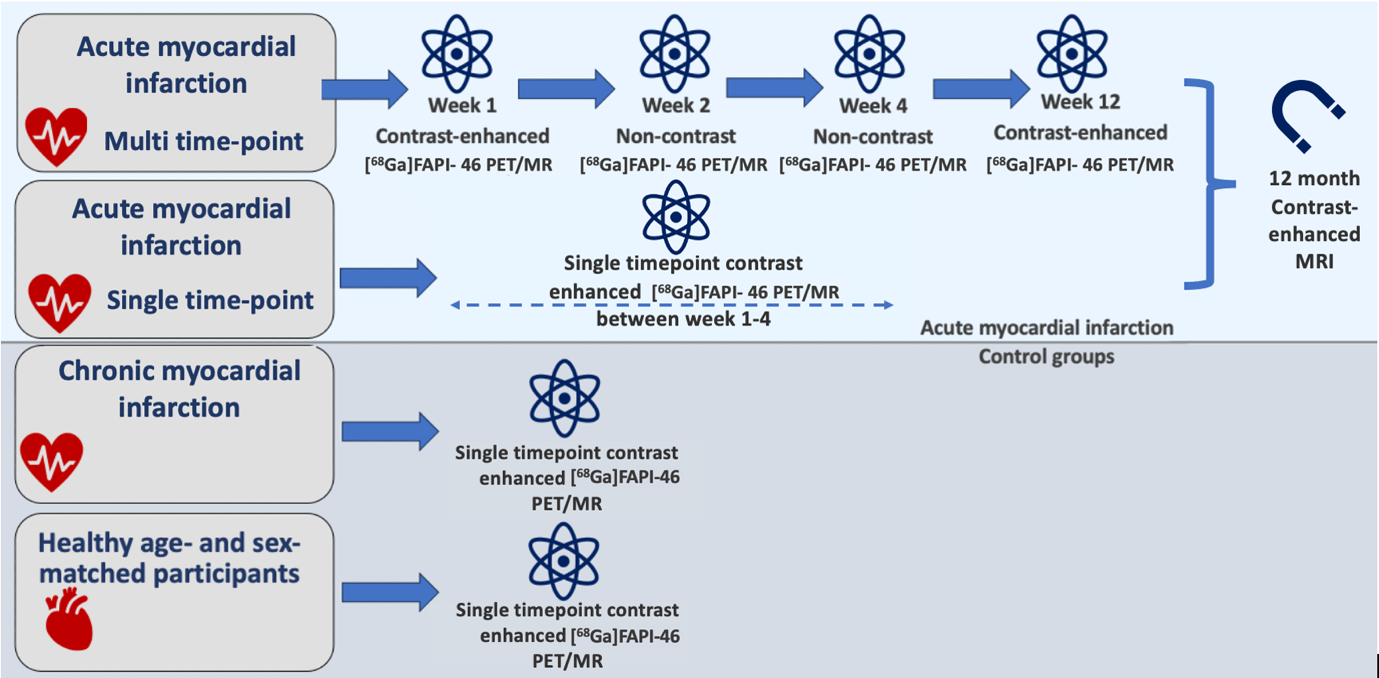


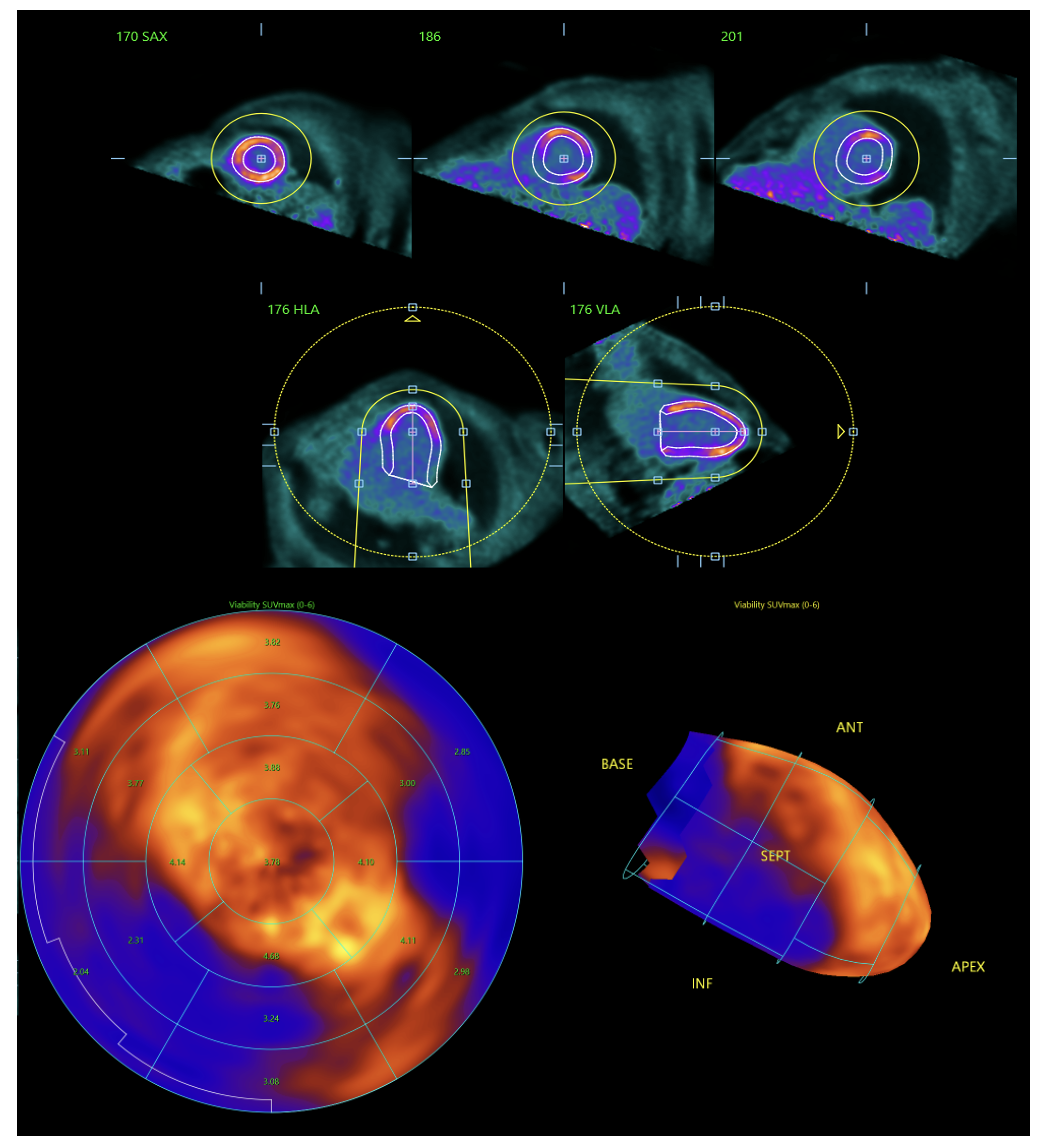


**Supplemental Figure 2: Semi-automated segmentation of the myocardial positron emission tomography signal.** Using QPET (Cedars-Sinai, Los Angeles), semi-automated segmentation of the myocardial positron emission tomography signal arising from the left ventricle is performed in 3 orthagonal planes using an adjustable left ventricular region of interest with a mask used to exclude all counts outside of the cardiac silhouette. Regional positron emission tomography uptake can then be quantified using the 17-segment myocardial model and a polar map generated.

**Supplemental Figure 3: Segmentation of the infarct,** **peri-infarct, and remote myocardial segments using FusionQuant.** Co-registered positron emission tomography and 3-dimensional short-axis stack magnetic resonance images were analysed using FusionQuant to define each of the above areas. The infarct was defined as the area of late gadolinium enhancement (green area on Panel B), and the peri-infarct as the area of [^68^Ga]FAPI-46 signal 1.5 times greater than maximum blood pool signal that extended beyond the late gadolinium enhancement (blue areas on Panel D). The remote myocardium was defined as the American Heart Association myocardial segment (Panel E) furthest away from the maximal [^68^Ga]FAPI-46 signal intensity (white area, Panel F). In this case demonstrating a right coronary artery-territory infarct, segment 10 showed maximal intensity and therefore segment 7 was selected as the remote myocardium (Panel F).

[^68^Ga]FAPI-46 = Gallium-68 Fibroblast Activation Protein Inhibitor-46


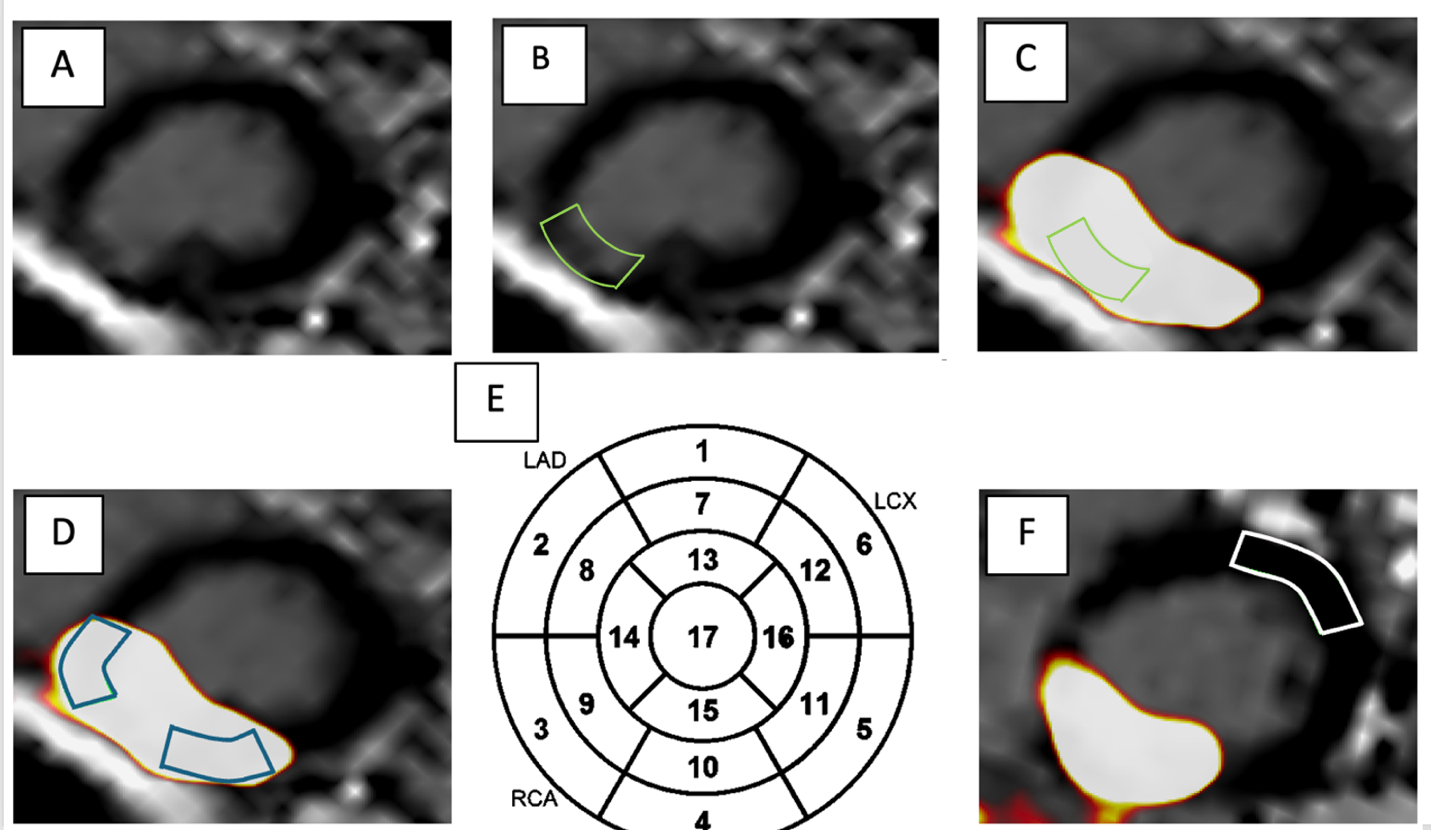
**Supplemental Figure 4. Intensity of myocardial fibroblast activation over time following myocardial infarction (global analysis)**

Panel A: illustrative PET/MR images from each timepoint; the images representing acute MI at each timepoint are taken from the same participant. The intensity of myocardial [^68^Ga]FAPI-46 uptake (TBR_max_, Panel B) was elevated in all patients with acute myocardial infarction at all timepoints compared to healthy volunteers and patients with chronic infarcts. [^68^Ga]FAPI-46 uptake was at its peak at weeks 1 and 2 following acute myocardial infarction, before starting to decline at weeks 4 and 12. Myocardial [^68^Ga]FAPI-46 uptake in those with chronic myocardial infarction remained elevated compared to the healthy volunteers.


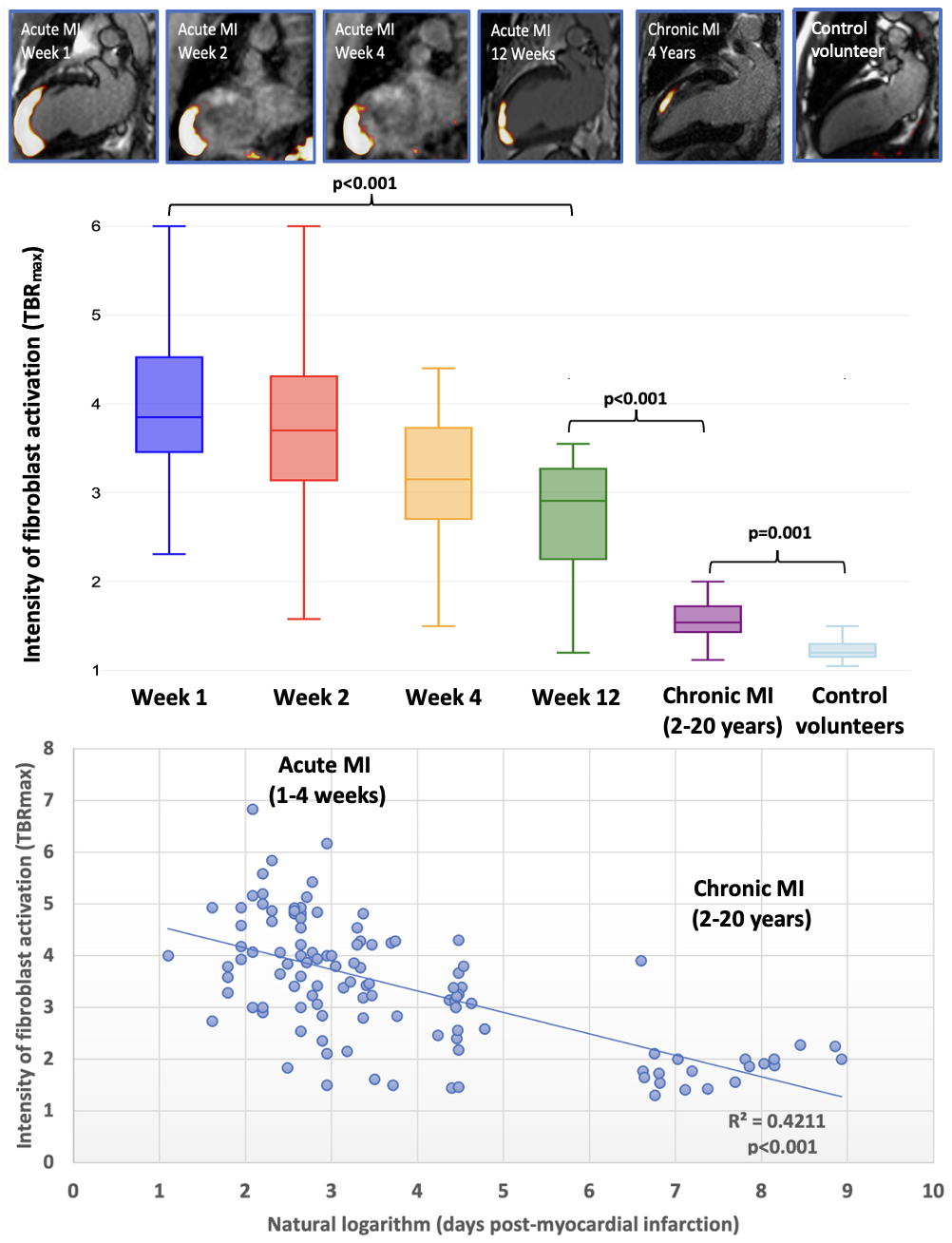


A

B


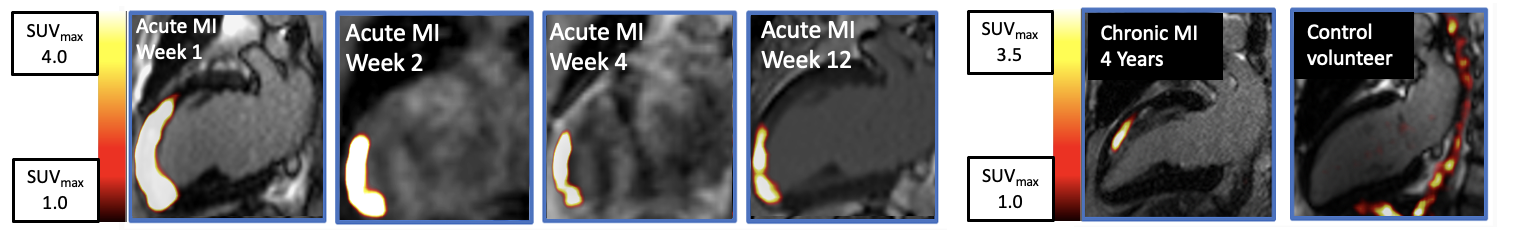


**Supplemental Figure 5: Right ventricular extension of fibroblast activation in myocardial infarction**

In 37.5% of all acute infarcts and in 60% of acute inferior infarcts, visible extension of [^68^Ga]FAPI-46 signal to the right ventricle was observed. In cases A+B, a 4-chamber magnetic resonance image with gadolinium contrast is displayed on the left, and in case C, a short-axis magnetic resonance image. These cases highlight the comparative difficulty in assessing late gadolinium enhancement in the right ventricular free wall compared with the clearer right ventricular [^68^Ga]FAPI-46 uptake in the image on the right.

[^68^Ga]FAPI-46 = Gallium-68 fibroblast activation protein inhibitor-46, CMR = Cardiac magnetic resonance, PET/MR = Positron emission tomography with magnetic resonance.


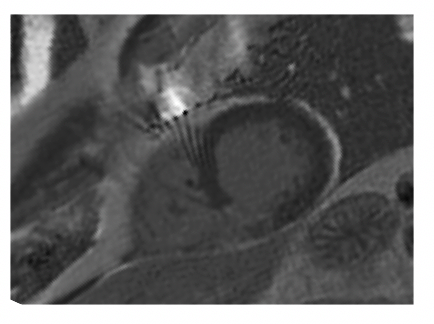

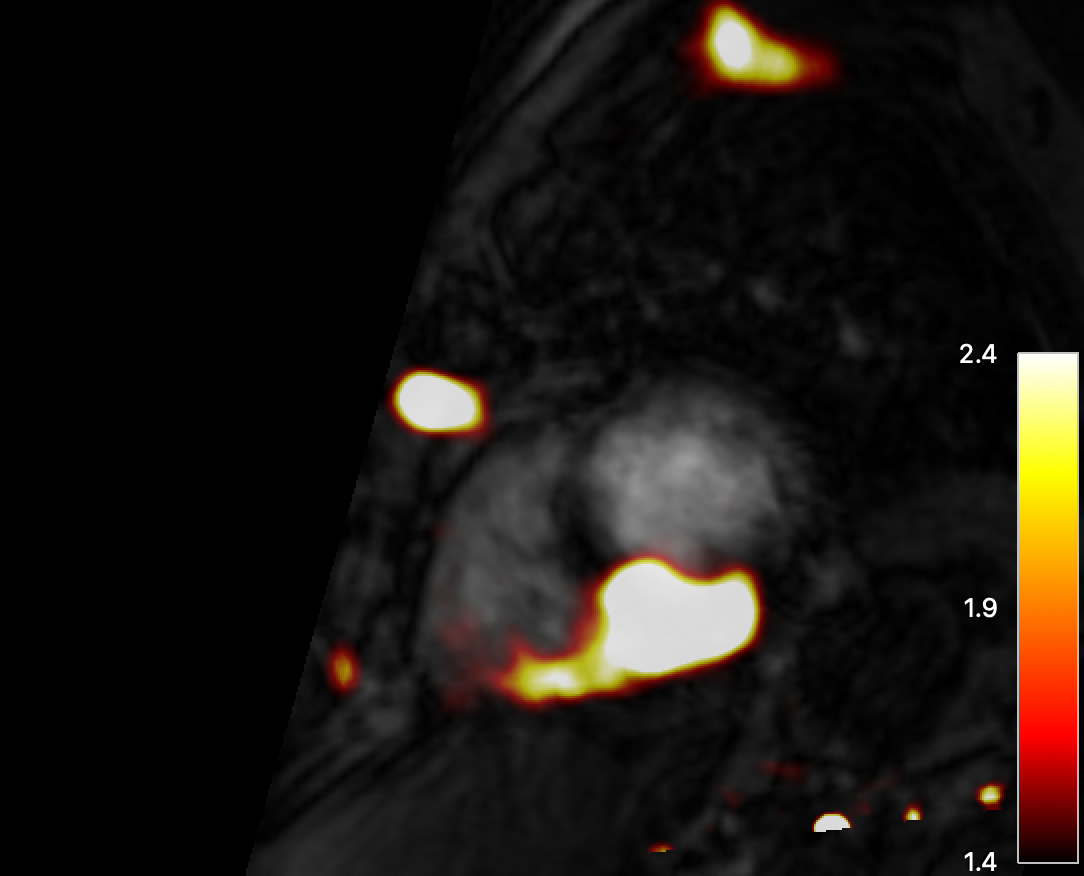

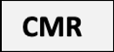

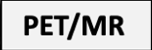

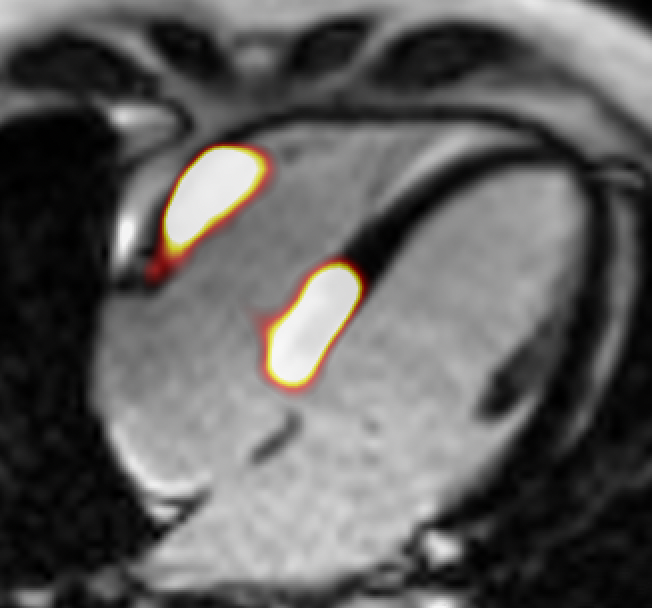

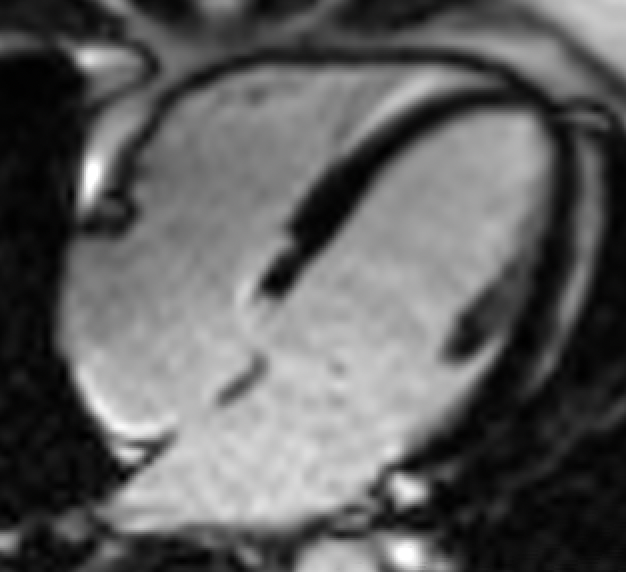

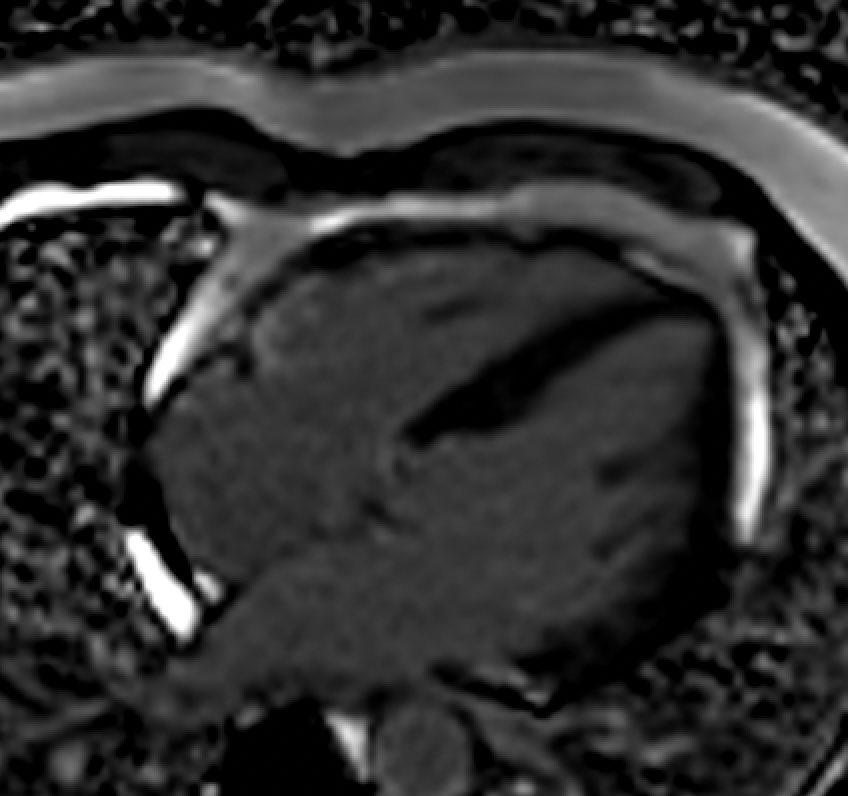

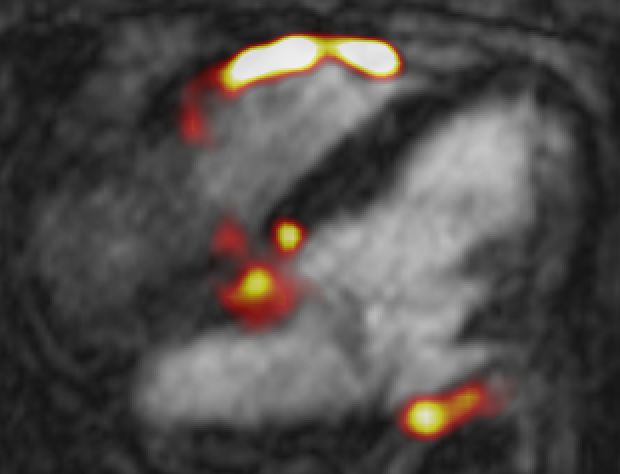

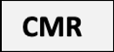

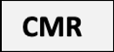

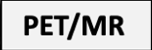

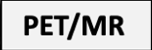


A

B

C


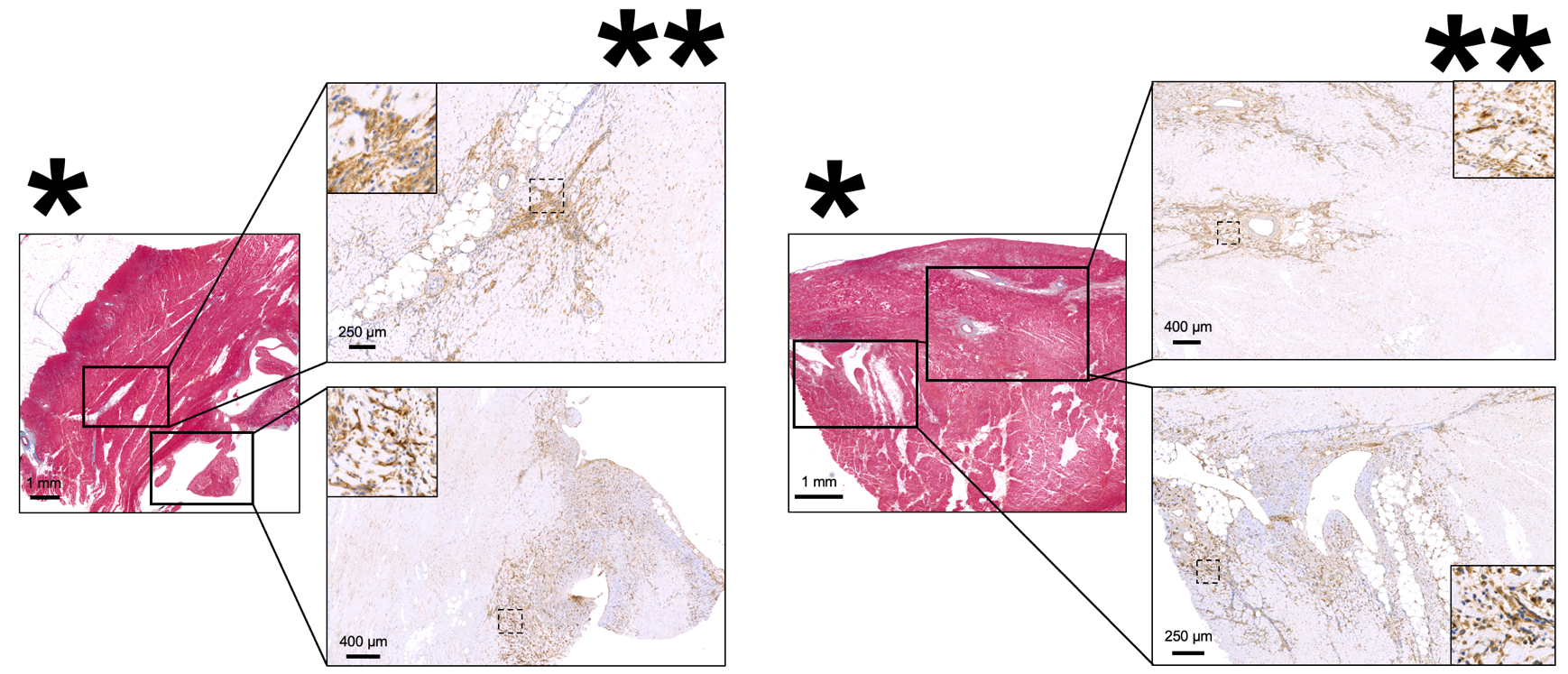
**
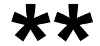
**
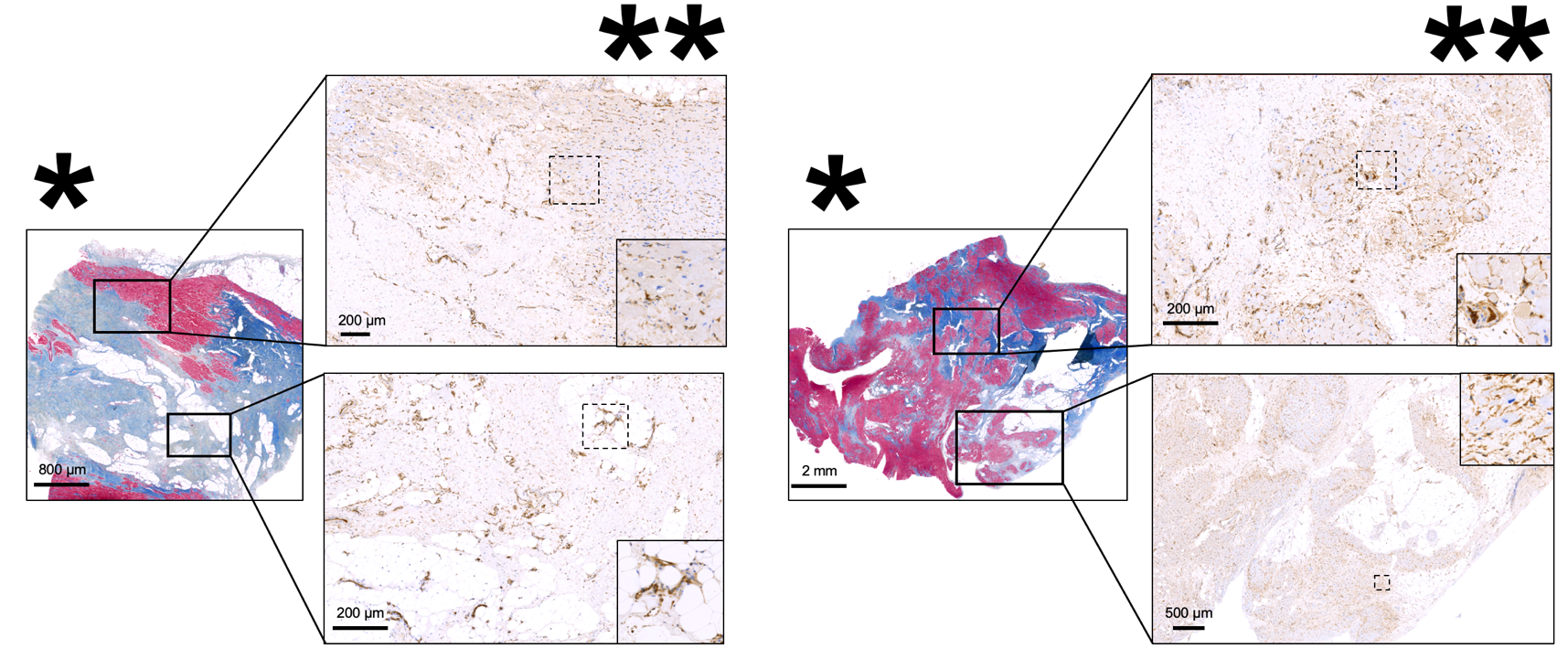

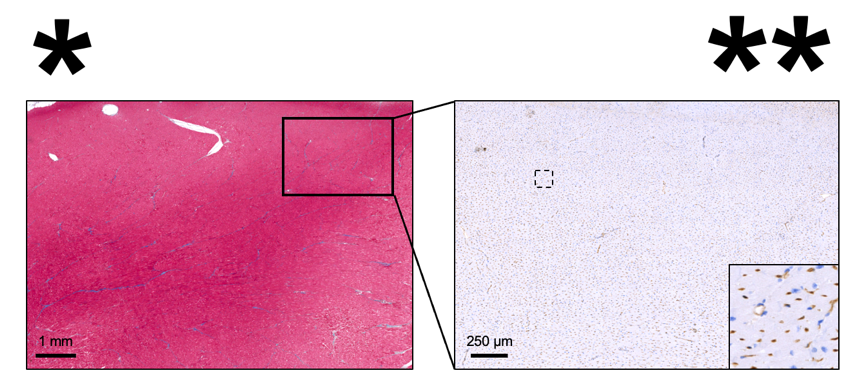


**Infarct zone**

**Infarct zone**

**Acute MI**

**Peri-infarct zone**

**Peri-infarct zone**

**Peri-infarct zone**

**Infarct zone**

**Infarct zone**

**Peri-infarct zone**

**Control**

**Chronic MI**

**Remote myocardium**


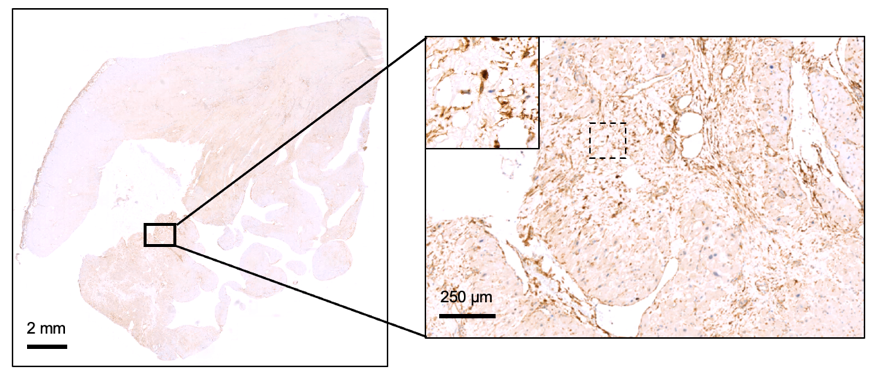


**Supplemental Figure 6: Histological Assessment of Fibroblast Activation Protein**

Fibroblast activation protein (FAP)-positive fibroblasts are identified on immunohistochemistry in regions of both acute and chronic myocardial infarction compared with negative immunohistochemistry in healthy myocardium. FAP-positive fibroblasts are also seen in the remote myocardium of those with acute myocardial infarction although fewer in number than in the infarct region.

* Masson’s Trichrome ** Immunohistochemistry for fibroblast activation protein-positive fibroblasts
